# Probabilistic analysis of agent-based opinion formation models

**DOI:** 10.1038/s41598-023-46789-3

**Published:** 2023-11-17

**Authors:** Carlos Andres Devia, Giulia Giordano

**Affiliations:** 1 Delft Center for Systems and Control, Delft University of Technology, 2628 CD Delft, The Netherlands; 2 Department of Industrial Engineering, University of Trento, 38123 Trento, Italy

**Keywords:** Applied mathematics, Computational science

## Abstract

When agent-based models are developed to capture opinion formation in large-scale populations, the opinion update equations often need to embed several complex psychological traits. The resulting models are more realistic, but also challenging to assess analytically, and hence numerical analysis techniques have an increasing importance in their study. Here, we propose the Qualitative Outcome Likelihood (QOL) analysis, a novel probabilistic analysis technique aimed to unravel behavioural patterns and properties of agent-based opinion formation models, and to characterise possible outcomes when only limited information is available. The QOL analysis reveals which qualitative categories of opinion distributions a model can produce, brings to light their relation to model features such as initial conditions, agent parameters and underlying digraph, and allows us to compare the behaviour of different opinion formation models. We exemplify the proposed technique by applying it to four opinion formation models: the classical Friedkin-Johnsen model and Bounded Confidence model, as well as the recently proposed Backfire Effect and Biased Assimilation model and Classification-based model.

## Introduction

Since French^1^, Harary^2,3^, and DeGroot^4^ proposed the first agent-based opinion formation models used to recreate and explain the mechanisms behind opinion formation in small groups, increasingly complex and realistic models have been proposed in the literature^5–9^ that often embed concepts from sociological and psychological research, including *attention*^10^, *cognitive dissonance*^11^, *bounded confidence*^12^, *polarity*^13^, *susceptibility*^14,15^, *leaders*^16,17^, *controversy*^18^, *stubbornness*^19,20^, *coevolving networks*^21,22^, *tolerance*^23^, *emotions*^24,25^, *weighted balance theory*^26^, *trust*^27,28^, *assimilation*^13,29^, *biases*^30–32^, *mass media*^33^, *curation algorithms and recommender systems*^34^.

Adding these features often results in rich and complex mathematical models, which are not in general amenable to theoretical analysis: the broad exploration of the model behaviour, properties, and capabilities relies on numerical approaches and simulation-based techniques^13,35–37^. A number of recently proposed techniques use numerical distributional measures to characterise opinion formation models and the opinion distributions they can produce: a histogram-based algorithm has been proposed to categorise opinion distributions and assess how the transitions between different opinion distribution categories in real populations are predicted by various agent-based models^38^; *Bias*, *Diversity*, and *Fragmentation* measures have been computed so as to classify opinion distributions and relate the model parameters with qualitative properties of the resulting opinions^13^; a graphical analysis can be employed to investigate how the model outcomes depend on the initial opinions, the agent parameters, and the underlying digraph^39^. Simulation-based techniques allow not only a thorough analysis of opinion formation models, but also a meaningful comparison of the behaviours of different models, and thus contribute to recent efforts towards common frameworks in which model behaviours can be studied, classified, and compared^38,40–42^.

In this paper, we propose a novel technique, based on probabilistic methodologies^43,44^, for the computational assessment of opinion formation models: the *Qualitative Outcome Likelihood (QOL) analysis*. Starting from partial information about the model parameters and initial conditions, we evaluate the probability that the opinion distribution after a chosen horizon belongs to one of the five qualitative opinion distribution categories proposed in^38^, namely: *Perfect consensus* (*PC*), when most of the agents have the same opinion; *Consensus* (*Co*), when most of the agents have a similar opinion; *Polarisation* (*Po*), when most of the agents are divided into two groups with distant opinions and the two groups have comparable size; *Clustering* (*Cl*), when most of the agents can be grouped in two or more clusters; and *Dissensus* (*Di*), when there is no discernible tendency. The algorithm that we use to sort an opinion distribution into one of the considered categories is described in the Supplementary Information and is a modified version to the one we previously presented^38^. Then, we construct **QOL Tables** that approximate the probability that the predicted opinion distribution belongs to one of the five possible categories, given *incomplete* information about the initial opinion distribution, the agent parameters, and the underlying digraph considered in the model. The probability is assessed by casting the possible opinion evolutions within the framework of a Bernoulli process, in which the Bernoulli trial is to determine whether the predicted opinion distribution belongs to a given category.

Being able to analyse the system in the absence of complete information is of special importance for opinion formation models, where measuring or estimating the system initial conditions and parameters is extremely challenging or even impossible. When there is no information at all on the initial opinion distribution *or* the agent parameters, the probability that the predicted opinion distribution belongs to the various qualitative categories is visualised by **QOL Figures**. Using **QOL Tables and Figures**, we can characterise the different qualitative opinion outcomes that a model can produce with suitably chosen parameters and gain insight into intrinsic properties of the model.

Our proposed technique can be applied to analyse any agent-based opinion formation model in which the opinion of each agent is a real number belonging to a bounded interval. The technique is exemplified by applying it to four opinion formation models: the classical Friedkin-Johnsen model^14,15^ and Bounded Confidence model^12^, as well as the recently proposed Classification-based model^45^ and Backfire Effect and Biased Assimilation model^46^. In all these models, opinions are represented by real values between $-1$ and $+1$.

In the Friedkin-Johnsen (FJ) model^14,15^, each agent has a single time-invariant parameter, $\lambda _i\in [0,1]$ for agent *i*, which captures its *susceptibility*. A completely susceptible agent ($\lambda _i=1$) updates its opinion to be the weighted average of the opinions of its neighbours (as in the French-DeGroot model^4^), while a completely non-susceptible agent ($\lambda _i=0$) sticks to its original opinion regardless of its neighbours’ opinions. Our QOL analysis reveals that the resulting qualitative opinion distribution primarily depends on the average population susceptibility ${\overline{\lambda }}$; when half of the agents are completely susceptible and half are completely non-susceptible, and when all agents have intermediate susceptibility, the outcome is qualitatively similar. Therefore, the opinion evolution at the population level can be understood without characterising individual agent traits: a global metric, such as the average susceptibility, already provides insightful information about the population behaviour.

Each agent *i* in the Bounded Confidence (BC) model^12^ has a confidence radius $r_i\in [0,2]$. At each time step, all agents update their opinions simultaneously. The new opinion of agent *i* is the average of all opinions in the population for which the difference with the previous opinion of agent *i* is less than or equal to $r_i$: every agent updates its opinion to the average of the opinions that are within its confidence radius. Although the BC model is commonly associated with clustering behaviour, our analysis indicates that this outcome is not the most likely, and in fact it requires specific initial conditions (polarised or evenly distributed initial opinions) and agent confidence radius (having a mean of approximately 0.5) to occur.

For the Classification-based (CB) model^45^, each agent has three inner traits whose strength is quantified by three parameters: $\alpha _i$, capturing conformism, which induces agent *i* to agree with its neighbours; $\beta _i$, capturing radicalism, which moves its opinion towards extreme values; $\gamma _i$, capturing stubbornness, which prevents its opinion from changing. The three parameters take values in the interval [0, 1] and sum up to 1. Our QOL analysis shows that the average radicalism ${{\bar{\beta }}}$ of the population is a good global indicator of the opinion behaviour: populations with high average radicalism tend to produce perfect consensus, whereas populations with low average radicalism, and hence mostly conformist and stubborn attitudes, tend to produce consensus, in that the predicted opinions are similar, but not the same.

The Backfire Effect and Biased Assimilation (BEBA) model^46^ can be seen as a generalisation of the French-DeGroot model that also includes: (1) backfire effect, which occurs when communication between agents *i* and *j* leads to their opinions becoming more distant than before their interaction; (2) biased assimilation, which is a form of homophily, a tendency to be more influenced by neighbours with similar opinions. The overall strength of both mechanisms is captured by the *entrenchment* parameter $\rho _i>0$ associated with each agent *i*. The QOL analysis shows that, in contrast with the FJ and CB model, and similarly to the BC model, for the BEBA model perfect consensus is the most likely outcome for almost all combinations of initial opinions, agent parameters, and underlying digraphs. In fact, both backfire effect and biased assimilation lead to perfect consensus: on the one hand, the backfire effect moves opinions to extremes and thus yields perfect consensus when the initial opinions have some bias (which is almost always the case, since the initial bias will result in one extreme opinion attracting significantly more agents than the other); on the other hand, biased assimilation moves opinions arbitrarily closer, also resulting in a tendency towards perfect consensus.

Perfect consensus is achieved by all four models, for a suitable choice of the initial conditions: the FJ model leads to perfect consensus whenever the initial opinions start from perfect consensus, since the model includes no mechanism to move opinions apart; the BC model produces perfect consensus, except when the initial opinions start polarised or evenly distributed and the set of agents’ confidence radii has approximate mean of 0.5 and variance of 0.8; the CB model yields perfect consensus when the average weight of radicalism is larger than 0.8, and thus strong radicalism moves the opinions to extremes, forming perfect consensus at one of the two extreme opinions, 1 or $-1$; and for the BEBA model, perfect consensus is almost certain for every initial condition (except when the population average entrenchment is higher than 0.8), because the interplay between the opposite effects of homophily and back-fire effect leads to the formation of two distinct opinion groups: the two groups are kept far apart by the back-fire effect and each group is kept together by homophily. Depending on the initial conditions, one of the two groups that are formed is likely to have significantly more agents than the other, which leads to classifying the resulting opinion distribution as perfect consensus (while it would be polarisation if the two agent groups had more comparable size).

## Methods

The **Qualitative Outcome Likelihood** (QOL) analysis technique relies on **QOL Tables** and **QOL Figures**. We call **QOL Tables** the collection of five tables containing the probabilities that the model yields a predicted opinion distribution qualitatively categorised as *PC*, *Co*, *Po*, *Cl*, *Di*, given approximate information about the initial opinions *and* agent parameters. Conversely, **QOL Figures** are a collection of ten figures that contain the probabilities of obtaining a certain qualitative outcome, when only approximate information about the initial opinions *or* the agent parameters is known.

In order to construct **QOL Tables** and **QOL Figures**, we first need to compute the **Qualitative Outcome Probability**, i.e., the probability that the predicted opinion distribution belongs to a specific qualitative category.

### Qualitative outcome probability

We consider a population with $N$ agents, where agent *i* is associated with its time-varying opinion $x_i\in [-1,1]$, $i\in {\mathcal {V}}=\{1, 2, \ldots , N\}$, with $x_i[k]$ denoting the opinion of agent *i* at time $k \in {\mathbb {N}}$. The opinion value represents the level of agreement or disagreement of the agent with a given statement: opinion values of $+1$, $-1$, and 0 represent complete agreement, complete disagreement, and indifference, respectively, while intermediate values correspond to less extreme opinions. The *opinion distribution* *x* is the set of all the opinions (non-ordered) of all the agents in the population. Each agent $i \in {\mathcal {V}}$ is also characterised by some parameters $p_i$; *p* denotes the set collecting all the parameter values (non-ordered) of all agents.

We then address the following question **Q**: *for a population of* $N$ *agents whose opinions evolve according to a given opinion formation model, if the initial opinion distribution belongs to a subset* ${\mathcal {O}}$ *of all possible initial opinion distributions, the agent parameters belong to a subset* ${\mathcal {P}}$ *of all possible agent parameters, and the underlying digraph belongs to a subset* ${\mathcal {N}}$ *of all possible digraphs with* $N$ *vertices, what is the probability that, after K time steps, the predicted opinion distribution can be categorised as perfect consensus (or consensus, or polarisation, or clustering, or dissensus)?*

This is a natural question to ask when only limited or incomplete information on the social system is available, which is the case in reality. When the information is not complete, the predicted opinions cannot be computed precisely, and one can at most evaluate the probability that they belong to a qualitative category. In some cases, knowing the exact initial opinions, agent parameters, and underlying digraph may not be very relevant (for instance, if the answer to **Q** with perfect consensus is $99\%$, then the qualitative outcome of the model is almost surely perfect consensus).

To provide a quantitative answer to question **Q**, we can proceed as follows:

1. Create three algorithms that randomly sample from the sets ${\mathcal {O}}$, ${\mathcal {P}}$, and ${\mathcal {N}}$ uniformly.
2. For a number of events $N_e$, do the following:
  - Randomly uniformly select an element from ${\mathcal {O}}$, ${\mathcal {P}}$, and ${\mathcal {N}}$ (an initial opinion distribution *x*, a set of agent parameters *p*, and an underlying digraph associated with weight matrix *W*),
  - evolve the system with the selected initial opinion distribution, agent parameter set, and underlying digraph according to the opinion model for the designated time steps,
  - categorise the predicted opinion distribution according to the sorting algorithm (based on^38^; for details, see the Supplementary Information): if the resulting category is the desired one, then the event is labelled as a success.
3. The previous sequence of $N_e$ events has the structure of a Bernoulli process, where the random variable is a success if the predicted opinion distribution belongs to the chosen category. Hence, the number of events $N_e$ and successes $N_s$ can be used to suitably approximate the probability that the predicted opinion distribution actually belongs to the desired category, i.e., to answer question **Q**.

Then, the probability ${\mathbb {P}}_B({\mathcal {O}}, {\mathcal {P}}, {\mathcal {N}}, K)$ that – if the initial opinion distribution belongs to the set ${\mathcal {O}}$, the agent parameter set belongs to the set ${\mathcal {P}}$, and the underlying digraph belongs to the set ${\mathcal {N}}$—the predicted opinion distribution after *K* time steps is categorised as $B\in \{PC, Co, Po, Cl, Di\}$ can be approximated by the Wilson score interval^43^:

1 $$\begin{aligned}{}&{\mathbb {P}}_B({\mathcal {O}}, {\mathcal {P}}, {\mathcal {N}}, K) \in [P-\delta , P+ \delta ] \quad \text {with probability 0.95,} \\&\text { given } P= \frac{N_s+ \frac{1}{2}z^2}{N_e+ z^2}, \quad \delta = \frac{z}{N_e+ z^2}\sqrt{\frac{(N_e-N_s)N_s}{N_e} + \frac{z^2}{4}}, \quad \text{ and } \quad z= 1.96, \nonumber \end{aligned}$$

where $z$ is the z-value for *95% confidence level*, while $N_e$ and $N_s$ are respectively the number of events and of successes of a Bernoulli process where the Bernoulli trial is the answer to the question: *Does the predicted opinion distribution belong to category* $B$?

The higher the number of events, the better the approximation: in fact, $\delta \rightarrow 0$ as $N_e\rightarrow \infty$. This procedure can be adjusted to account for different forms of available information. For instance, if some correlation between initial opinion assignation and agent parameters is known, it can be added as a constraint to the process. The only requirement is that the random variables are independent and identically distributed, which means that every possible initial configuration should be equally likely to be chosen so as to be evolved and produce predicted opinions that are then categorised.

In Eq. (1), ${\mathbb {P}}_B$ is the **real** probability that answers question **Q**, while $P$ is a value that we can compute to **approximate** the real probability with a desired confidence level. We choose the z-value for the 95% confidence level so that the probability ${\mathbb {P}}_B({\mathcal {O}}, {\mathcal {P}}, {\mathcal {N}}, K)$ of the Bernoulli process belongs to the interval $[P-\delta , P+ \delta ]$ with a probability of 0.95 (namely, the inclusion ${\mathbb {P}}_B({\mathcal {O}}, {\mathcal {P}}, {\mathcal {N}}, K) \in [P-\delta , P+ \delta ]$ in Equation (1) is true with probability $95\%$). Other confidence levels may be chosen by using the corresponding z-value.

For all the simulations in this paper, the number of events $N_e$ is at least 10000. Hence, for any possible number of successes $N_s\in \{0, 1, \ldots , N_e\}$, the uncertainty $\delta$ is less than 0.01 (see Fig. 1 in the Supplementary Information). Hence, for all the results we show,  $|{\mathbb {P}}_B- P|<0.01$ with 95% probability, where $P$ is as defined in Eq. (1). For simplicity, we will shortly say that we are ‘computing the probability ${\mathbb {P}}_B$’ when actually we are ‘computing the value $P$ such that $|{\mathbb {P}}_B- P|<0.01$ with 95% probability’.

Equation (1) is useful to answer questions like **Q**, where the sets ${\mathcal {O}}$, ${\mathcal {P}}$, and ${\mathcal {N}}$ are given. However, a systematic application of Eq. (1) over a family of sets of initial opinion distributions, a family of sets of agent parameter sets, and a family of sets of underlying digraphs can provide further insight into the model properties and behaviour patterns. Here, we just present one possible application of this systematic approach, aimed at investigating the relation between ${\mathbb {P}}_B$ and the initial opinion distribution and agent parameter set; but the approach could be adapted to investigate the relation between the model outcome and properties of the digraph, or other properties of the model.

### Qualitative outcome likelihood tables and figures

Before analysing models through the systematic application of Eq. (1), we need to introduce some notation and concepts.

#### Families of opinion distribution sets and sets of agent parameter sets

Every opinion distribution $x \in [-1, 1]^N$ can be related to two values, the average ${\overline{x}}$ and the average of the absolute values $\overline{|x|}$. Then, an opinion distribution can be represented as a point in the Cartesian plane, whose abscissa is $\overline{|x|}$ and whose ordinate is ${\overline{x}}$, resulting in the **Agreement Plot**^39^.

We denote by $\{{\mathcal {O}}_i\}$ an ordered *family* of opinion distribution sets. The generic set ${\mathcal {O}}_i= {\mathcal {O}}_i\left( ({\overline{x}})_i, (\overline{|x|})_i, \epsilon _o\right)$ of opinion distributions is associated with three numbers, $({\overline{x}})_i$, $(\overline{|x|})_i$ and $\epsilon _o>0$, so that a generic opinion distribution $\xi$ belongs to ${\mathcal {O}}_i$ if

2 $$\begin{aligned} \left( {\overline{\xi }} - ({\overline{x}})_i\right) ^2 + \left( \overline{|\xi |} - (\overline{|x|})_i\right) ^2\le \epsilon _o^2. \end{aligned}$$

Agents are typically characterised by time-invariant parameters that capture their psychological traits. For instance, the generic agent $i\in {\mathcal {V}}$ is characterised by *susceptibility* $\lambda _i \in [0, 1]$ in the FJ model, by the triple $\psi _i=(\alpha _i, \beta _i, \gamma _i) \in [0,1]^3$ such that $\alpha _i+\beta _i+\gamma _i=1$ in the CB model, by *entrenchment* $\rho _i>0$ in the BEBA model, and by a *confidence radius* $r\in [0,2]$ in the BC model. The set of all agent parameters of a whole population is called the *agent parameter set* and generically denoted *p* (for the FJ, CB, BEBA, and BC models we denote the agent parameter set as $\lambda$, $\psi$, $\rho$, and *r*, respectively).

Every agent parameter set can also be related to two values, chosen depending on the specific model, which are suitably chosen functions of *p* that we generically denote as $f_a(p)$ and $f_b(p)$. We consider: for the FJ model, the average ${\overline{\lambda }}$ and the variance $\sigma (\lambda )$ of the susceptibility; for the CB model, the average radicalism ${\overline{\beta }}$ and average stubbornness ${\overline{\gamma }}$; for the BEBA model, the average ${\overline{\rho }}$ and the variance $\sigma (\rho )$ of the entrenchment; for the BC model, the average ${\overline{r}}$ and the variance $\sigma (r)$ of the confidence radius. Then, an agent parameter set can be represented as a point in the Cartesian plane, whose abscissa is $f_a(p)$ and whose ordinate is $f_b(p)$, resulting in the **Parameter Plot**.

We denote by $\{{\mathcal {P}}_j\}$ an ordered *family* of sets of agent parameter sets. The generic set ${\mathcal {P}}_j= {\mathcal {P}}_j(p_{a,j}, p_{b,j}, \epsilon _p)$ of agent parameter sets is associated with three numbers, $p_{a,j}$, $p_{b,j}$ and $\epsilon _p>0$, so that a generic agent parameter set $\pi$ belongs to ${\mathcal {P}}_j$ if

3 $$\begin{aligned} (f_a(\pi ) - p_{a,j})^2 + (f_b(\pi ) - p_{b,j})^2\le \epsilon _p^2, \end{aligned}$$

where the functions $f_a$ and $f_b$ are as previously described.

#### Implementation

Having defined the families $\{{\mathcal {O}}_i\}$ and $\{{\mathcal {P}}_j\}$ and how their elements relate to the points $({\overline{x}}, \overline{|x|})$ and $(p_a, p_b)$, we can now describe the procedure to create **QOL Tables** and **QOL Figures**. In the following, this systematic approach is explained resorting to the FJ model as a case study.

We focus on assessing how ${\mathbb {P}}_B$ changes depending on the global features of the initial opinion distribution *x* and the agent parameter set *p* (which corresponds to the agent susceptibility $\lambda$ for the FJ model). The set of underlying digraphs ${\mathcal {N}}$ is fixed, and includes 1000 digraphs with Small-World topology (for more details, see the Supplementary Information), and the evolution horizon is fixed to $K=50$. Then, the probability becomes a function of ${\mathcal {O}}$ and ${\mathcal {P}}$ only: ${\mathbb {P}}_B={\mathbb {P}}_B\big ({\mathcal {O}}, {\mathcal {P}}\big )$.

**Figure 1:**
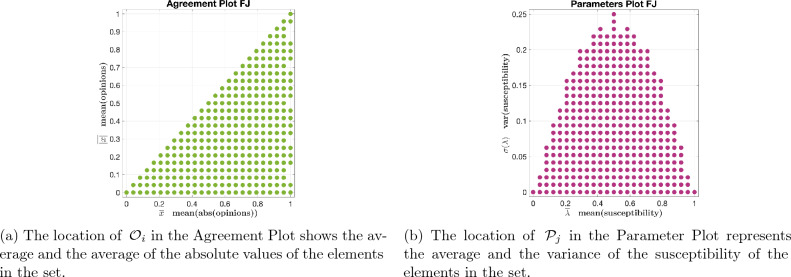
Sets ${\mathcal {O}}_i$ and ${\mathcal {P}}_j$ used in the QOL analysis of the Friedkin-Johnsen model. Taking a point in panel (**a**), with coordinates $({\overline{x}}, \overline{|x|})$, and a point in panel (**b**), with coordinates $({\overline{\lambda }}, \sigma (\lambda ))$, we can assess the probability ${\mathbb {P}}_B$ that a population starting from an initial opinion distribution with average ${\overline{x}}$ and average of the absolute values $\overline{|x|}$, whose susceptibility has average ${\overline{\lambda }}$ and variance $\sigma (\lambda )$, produces a predicted opinion distribution categorised as $B$, with $B\in \{PC, Co, Po, Cl, Di\}$.

We assess the qualitative behaviour of the FJ model by considering a *family* of initial opinion distribution sets that contains 318 different sets, $\{{\mathcal {O}}_i\}_{i=1}^{318}$, and a *family* of sets of agent parameter sets that contains 371 different sets, $\{{\mathcal {P}}_j\}_{j=1}^{371}$. In particular:

- Every opinion distribution $\xi \in {\mathcal {O}}_i$ meets the condition in Eq. (2) with $\epsilon _o = 0.05$. Figure 1a shows a representation of the 318 considered sets ${\mathcal {O}}_i$, where each green dot corresponds to a set ${\mathcal {O}}_i$, $i=1,\ldots ,318$, and its location in the Cartesian plane is determined, based on Eq. (2), by the corresponding values of $({\overline{x}})_i$ and $(\overline{|x|})_i$.
- Every set of agent parameters $\pi \in {\mathcal {P}}_j$ with average ${\overline{\pi }}$ and variance $\sigma (\pi )$ meets the condition in Eq. (3) with $\epsilon _p = 0.05$, $p_{a,j}=({\overline{\lambda }})_j$, $f_a(\pi ) = {\overline{\pi }}$, $p_{b,j}=(\sigma (\lambda ))_j$, and $f_b(\pi )=\sigma (\pi )$. Figure 1b shows a representation of the 371 considered sets ${\mathcal {P}}_j$, where each magenta dot corresponds to a set ${\mathcal {P}}_j$, $j=1,\ldots ,371$, and its location in the Cartesian plane is determined, based on Eq. (3), by the corresponding values of $({\overline{\lambda }})_j$ and $(\sigma (\lambda ))_j$.

Thanks to this characterisation, we can denote with

4 $$\begin{aligned} {\mathbb {P}}_B={\mathbb {P}}_B\big ({\mathcal {O}}, {\mathcal {P}}\big )= {\mathbb {P}}_B\big ({\overline{x}}, \overline{|x|}, {\overline{\lambda }}, \sigma (\lambda )\big ) \end{aligned}$$

the probability that, starting from an initial opinion distribution with average ${\overline{x}}$ and average absolute value $\overline{|x|}$, for a population whose susceptibility has average ${\overline{\lambda }}$ and variance $\sigma (\lambda )$ and whose opinions evolve according to the FJ model over a strongly connected Small-World network digraph in ${\mathcal {N}}$, the predicted opinion distribution after $K=50$ time steps belongs to the category $B$, with $B\in \{PC, Co, Po, Cl, Di\}$.

Any pair of points (one green and one magenta) in Fig. 1a and b represents a pair of sets ${\mathcal {O}}_i$ and ${\mathcal {P}}_j$, which can be associated with a number in the interval [0, 1] that quantifies the probability ${\mathbb {P}}_B={\mathbb {P}}_B({\mathcal {O}}_i, {\mathcal {P}}_j)= {\mathbb {P}}_B\big (({\overline{x}})_i, (\overline{|x|})_i, ({\overline{\lambda }})_j, (\sigma (\lambda ))_j\big )$. Since ${\mathbb {P}}_B$ depends on four parameters, it cannot be plotted directly.

**Figure 2:**
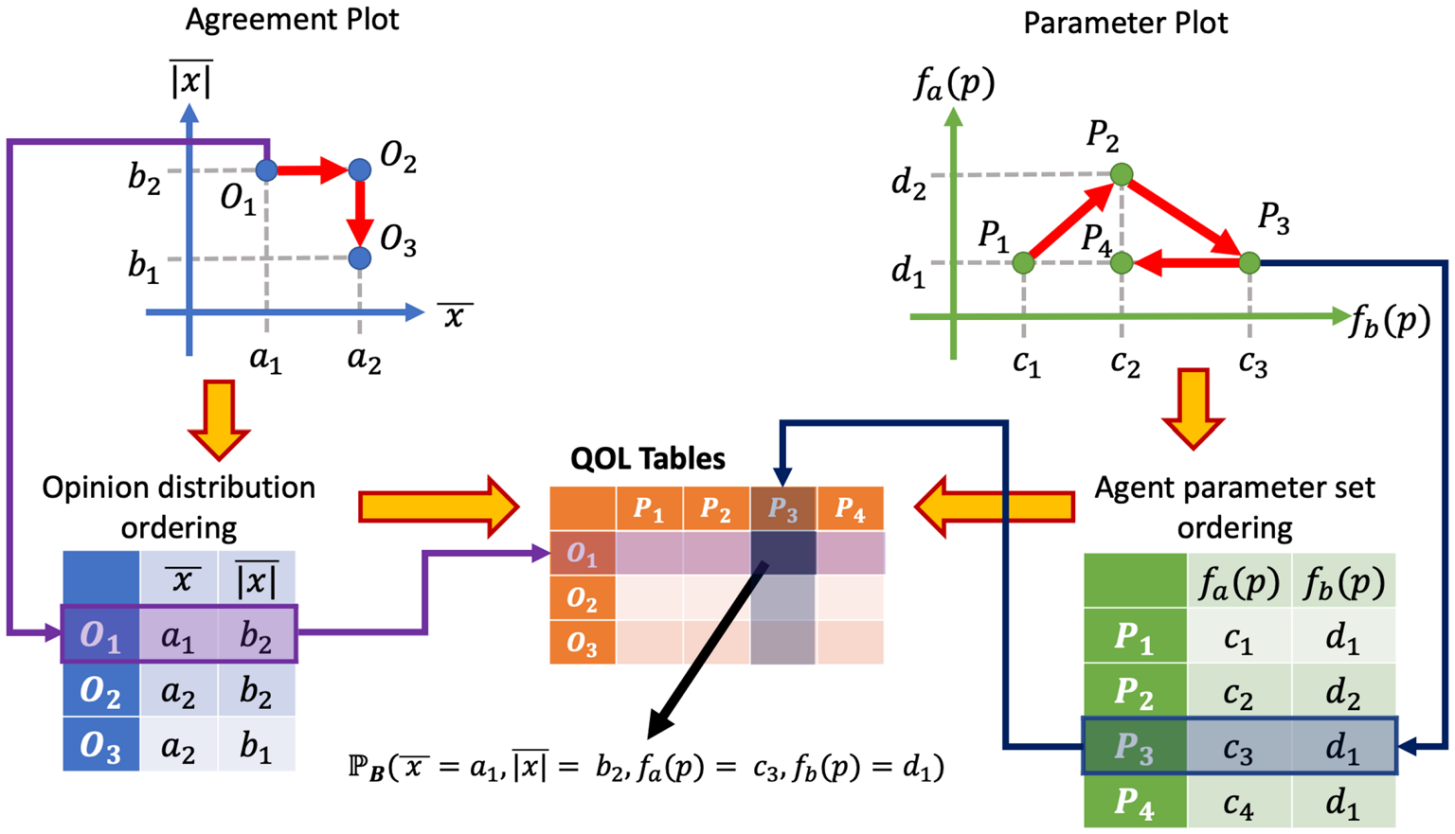
Construction of **QOL Tables**. Each of the points in the Agreement Plot has coordinates $\big (({\overline{x}})_i, (\overline{|x|})_i\big )$ corresponding to a set ${\mathcal {O}}_i$: they are ordered as $O_1, O_2, O_3$ following the red arrows. Analogously, each of the points in the Parameter Plot has coordinates $(p_{a,j}, p_{b,j})$ corresponding to a set ${\mathcal {P}}_j$: they are ordered as $P_1, P_2, P_3, P_4$ following the red arrows. Then, the **QOL Tables** are constructed, whose rows are associated with points in the Agreement Plot and whose columns are associated with points in the Parameter Plot, in the corresponding order. Therefore, the cell in position (*i*, *j*) in the **QOL Tables** is associated with point ${\mathcal {O}}_i = \big ( ({\overline{x}})_i, (\overline{|x|})_i\big )$ in the Agreement Plot and with point ${\mathcal {P}}_j = (p_{a,j}, p_{b,j})$ in the Parameter Plot, and contains a value between 0 and 1 that represents the probability ${\mathbb {P}}_B$.

However, as shown by the schematic example in Fig. 2, by suitably ordering the points in Fig. 1a and b, all the probabilities ${\mathbb {P}}_B$ for different choices of $({\overline{x}}, \overline{|x|})$ and of $({\overline{\lambda }}, \sigma (\lambda ))$ can be arranged in a Table. Since $B\in \{PC, Co, Po, Cl, Di\}$, this procedure actually leads to five different tables, one per qualitative category of predicted opinion distribution, which are collectively called the Qualitative Outcome Likelihood Tables (**QOL Tables**).

In the FJ model case, each of the five **QOL Tables** has 318 rows (the number of points in Fig. 1a) and 371 columns (the number of points in Fig. 1b), and each of its 117978 cells contains a number between 0 and 1. Due to the huge number of cells, instead of showing the value of each cell, it is more practical and insightful to represent the tables visually by an image, where the colour of each cell depends on its value: probabilities near 0 (respectively, 0.5, and 1) have a light blue (resp. purple, and orange) colour. This colour convention for the probability values is used for all the QOL analysis and can be seen in the colour-bars of the figures.

**Figure 3:**
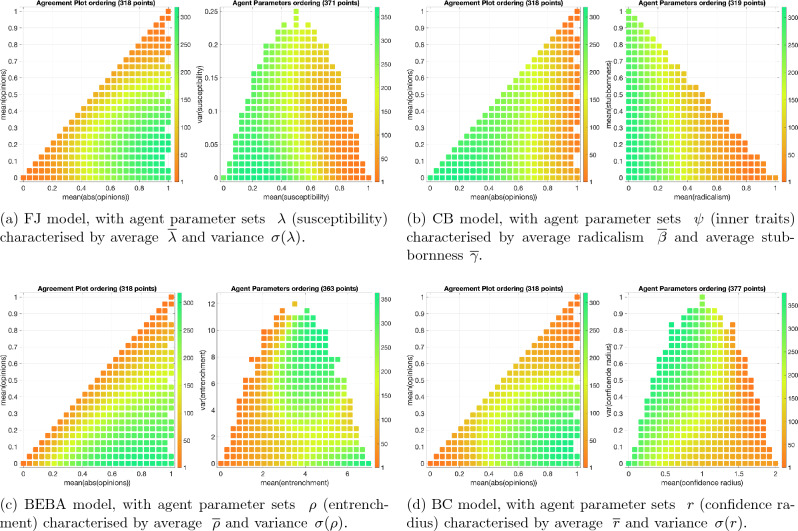
Ordering of the points in the Agreement Plot and in the Parameter Plot for the four considered models. Each point $({\overline{x}}, \overline{|x|})$ in the Agreement Plot corresponds to a set ${\mathcal {O}}$ of initial opinion distributions. Each point $(p_a, p_b)$ in the Parameter Plot is related to a set ${\mathcal {P}}$ of agent parameters. The order of the points, indicated by the colorbars, is used when plotting the corresponding **QOL Tables** as explained in Fig. 2: Agreement Plot points are associated with rows, Parameter Plot points with columns of the **QOL Tables**. This leads to an intuitive representation: for instance, cells near the bottom left corner of the **QOL Tables** correspond to high rows and low columns, which for the FJ model, according to the order in Fig. 3a, are associated with societies with high ${\overline{x}}$ and low $\overline{|x|}$ (i.e., initially polarised), and with high ${\overline{\lambda }}$ and low $\sigma (\lambda )$ (i.e., very susceptible).

**Figure 4:**
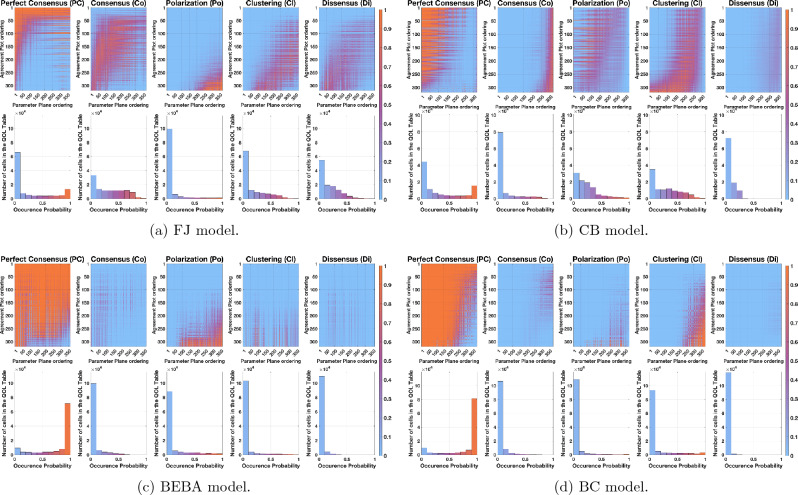
**QOL Tables** and corresponding histograms for the FJ, CB, BEBA, and BC models. First row: **QOL Tables** for $B\in \{PC, Co, Po, Cl, Di\}$. The rows corresponds to points $({\overline{x}}, \overline{|x|})$ in the Agreement Plot and the columns to points $(p_a, p_b)$ in the Parameter Plot, both ordered as in Fig. 3: each cell is associated with a set of initial opinion distributions and a set of agent parameter sets. The cell colour represents the probability ${\mathbb {P}}_B$, computed as per Eq. 1 (with 10000 samples), that the predicted opinion distribution after 50 time steps is categorised as $B\in \{PC, Co, Po, Cl, Di\}$. For details on the construction of **QOL Tables**, see Fig. 2. Second row: histograms corresponding to the **QOL Tables**, visualising the number of cells in the table whose probability value ${\mathbb {P}}_B$ is in a given interval; the bar colour reflects the corresponding probability.

The order of the points in Fig. 1a and b is directly related to the order of rows and columns in the **QOL Tables**. Hence, a suitable order may reveal patterns, such as clusters or regions in the table with particularly high or low probability. One can also plot the histogram of the cell values in the **QOL Tables**, which is invariant with respect to row and column permutations and represents the overall probability that a certain predicted category is obtained. For the FJ model, Fig. 3a shows the order of points, while Fig. 4a shows the corresponding visualisations of the **QOL Tables** and their corresponding histograms.

**Figure 5:**
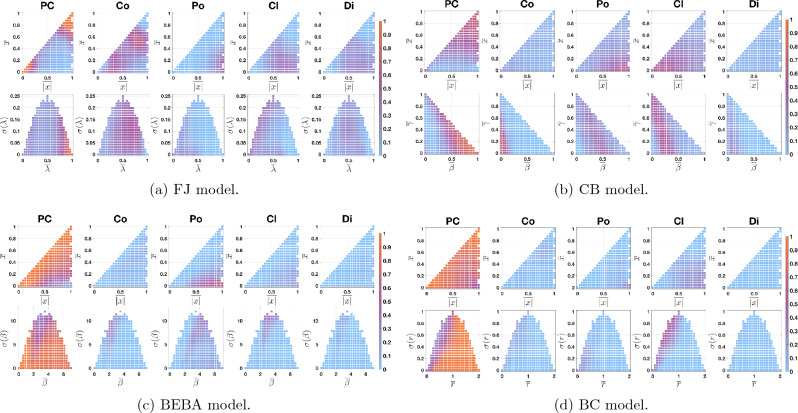
When no information on agent parameters (respectively, initial opinions) is available, the probabilities ${\mathbb {P}}_B$ that the predicted opinion distribution after 50 time steps is categorised as $B\in \{PC, Co, Po, Cl, Di\}$ can be visualised directly in the Agreement Plot (respectively, in the Parameter Plot), as shown in the first (respectively, second) row of each panel. For each dot, the probability ${\mathbb {P}}_B$ is computed as per Eq. (1) with a different number of samples. The number of samples for each point in the first row is 10,000 times the number of Agent Parameter points shown in Fig. 3 (in the right plots of each panel). The number of samples for each point in the second row is 3,180,000, since that is 10,000 times the number of points in the Agreement Plot in Fig. 3 (in the left plots of each panel).

Assume now that no information at all is available about the initial opinion distribution (respectively, about the agent parameters). Then, we can perform the analysis by taking ${\mathcal {O}}$ (resp. ${\mathcal {P}}$) as the set of all possible initial opinion distributions (resp. all possible agent parameter sets). In this case, ${\mathbb {P}}_B$ depends only on ${\overline{\lambda }}$ and $\sigma (\lambda )$ (resp. on ${\overline{x}}$ and $\overline{|x|}$), and therefore the resulting probabilities can be visualised directly in the Parameter Plot (resp. Agreement Plot). The resulting ten **QOL Figures** show the probabilities ${\mathbb {P}}_B$ for $B\in \{PC, Co, Po, Cl, Di\}$ when only incomplete information on *either* the initial opinions *or* the agent parameters is available. Figure 5a shows the results for the FJ model. **QOL Figures** can be seen as a visualisation of the **QOL Tables** when there is only one set of initial opinion distributions (respectively, of agent parameter sets): since there is only one row (resp. column), the table values can be directly represented in the Parameter Plot (resp. Agreement Plot). The resulting figures are invariant to the order of opinion distribution sets and of sets of agent parameter sets.

To summarise, the complete QOL analysis involves a three-step process:

1. **Agreement Plot and Parameter Plot Ordering**: To analyse the model, define the family of possible initial opinion distribution sets $\{{\mathcal {O}}_i\}$, where each set ${\mathcal {O}}_i= {\mathcal {O}}_i\left( ({\overline{x}})_i, (\overline{|x|})_i, \epsilon _o\right)$ is characterised according to Eq. (2), and the family of possible sets of agent parameter sets $\{{\mathcal {P}}_j\}$, where each set ${\mathcal {P}}_j= {\mathcal {P}}_j(p_{a,j}, p_{b,j}, \epsilon _p)$ is characterised according to Eq. (3). Order them suitably (a suitable order is one that can be easily interpreted and that results in **QOL Tables** with clear regions of high probability; in this paper, the ordering was found by numerically exploring various possibilities).
2. **Compute and analyse the QOL Tables**: For each pair of opinion distribution and agent parameter sets $({\mathcal {O}}_i, {\mathcal {P}}_j)$, compute the probability ${\mathbb {P}}_B({\mathcal {O}}_i, {\mathcal {P}}_j)$ that the predicted opinion distribution is categorised as $B$ for $B\in \{PC, Co, Po, Cl, Di\}$; the probability in Eq. (4) is approximated by the value *P* in Eq. (1). This provides the value associated with row *i* and column *j* in the corresponding table. Then, plot the table by colour-coding the cells depending on their value and analyse them taking into account the row and column order interpretation. It is also possible to plot and analyse the table histograms, which are independent of the order chosen at the previous step 1.
3. **Compute and analyse the QOL Figures**: If no information on the initial opinion distribution is available, the probability ${\mathbb {P}}_B$ depends only on the considered set of agent parameter sets ${\mathcal {P}}_j$; therefore, the probability values can be colour-coded in the Parameter Plot of the family $\{{\mathcal {P}}_j\}$. Analogously, if no information on the agent parameters is available, the probability ${\mathbb {P}}_B$ depends only on the considered set of opinion distributions ${\mathcal {O}}_i$; therefore, the probability values can be colour-coded in the Agreement Plot of the family $\{{\mathcal {O}}_i\}$.

If no information is available on *both* the initial opinions and the agent parameters, then one can only consider ${\mathcal {O}}$ as the set of all possible opinion distributions and ${\mathcal {P}}$ as the set of all possible agent parameter sets, which yields **QOL Tables** with a single cell, i.e. a single probability, for each table. Table 1 reports the five probabilities for each of the considered models.

The procedure described above requires that the opinion $x_i$ of each agent is scalar-valued and belongs to the interval $[-1, 1]$. However, our analysis can be adapted to models for which this is not the case. If the opinions are still unidimensional and bounded (for instance, in the interval [0, 1] instead), our proposed procedure can be applied by either normalising the opinions to be in the $[-1, 1]$ interval or suitably adapting the definition of the considered opinion categories (*Perfect Consensus*, *Consensus*, *Polarisation*, *Clustering*, and *Dissensus*). If the opinions are not bounded, or are multidimensional, then the procedure can be applied after having suitably redefined the opinion categories. In models with multidimensional opinions, where all dimensions are bounded, it is also possible to perform our proposed probabilistic analysis in each dimension independently.

The procedure to compute **QOL Tables** and **QOL Figures** is summarised in the following pseudo-code:

1. **Input**: the number of agents $N$; the simulation horizon *K*; a collection $\Upsilon =\big \{\big ({\overline{x}}_i, \overline{|x|}_i\big )\big \}_{i=1}^{\mathcal {I}}$ representing ${\mathcal {I}}$ points in the Agreement Plane; a collection $\Psi =\big \{\big ((p_a)_j, (p_b)_j\big )\big \}_{j=1}^{\mathcal {J}}$ representing ${\mathcal {J}}$ points in the Parameter Plane; a collection of interaction networks of suitable size ${\mathcal {N}}$; a number of events $N_e$; tolerance values $\epsilon _o$ and $\epsilon _p$; and a colour map *CM* for the interval [0, 1].
2. Initialise five empty tables $T_{PC}$, $T_{Co}$, $T_{Po}$, $T_{Cl}$, $T_{Di}$ with ${\mathcal {I}}$ rows and ${\mathcal {J}}$ columns.
3. For each point $\big ({\overline{x}}_i, \overline{|x|}_i\big )$ in $\Upsilon$ compute a set of initial opinion distributions ${\mathcal {O}}_i$ satisfying Equation (2): the collection of these sets forms the ordered family $\{{\mathcal {O}}_i\}_{i=1}^{\mathcal {I}}$.
4. For each point $\big ((p_a)_j, (p_b)_j\big )$ in $\Psi$ compute a set of agent parameter sets ${\mathcal {P}}_j$ satisfying Equation (3): the collection of these sets forms the ordered family $\{{\mathcal {P}}_j\}_{j=1}^{\mathcal {J}}$.

5. For each pair of indices (*i*, *j*) where $i=1, \dots , {\mathcal {I}}$ and $j=1, \dots , {\mathcal {J}}$ do the following:
  - Initialise five counters $N_{s,i,j,PC}=0$, $N_{s,i,j,Co}=0$, $N_{s,i,j,Po}=0$, $N_{s,i,j,Cl}=0$, $N_{s,i,j,Di}=0$: these are the success counters for each of the Bernoulli trials for the pair (*i*, *j*).
  - For $N_e$ iterations do:
  (i) Take a *randomly selected* opinion distribution $\xi \in {\mathcal {O}}_i$, a *randomly selected* set of agent parameters $\pi \in {\mathcal {P}}_j$, and a *randomly selected* underlying digraph $G\in {\mathcal {N}}$.
  (ii) Evolve the model with agent parameters $\pi$, initial opinions $\xi$, over the digraph *G* for *K* time steps. Call the predicted opinions $\xi _f$.
  (iii) Classify the predicted opinions $\xi _f$ into one of the five possible categories *Perfect Consensus* (*PC*), *Consensus* (*Co*), *Polarisation* (*Po*), *Clustering* (*Cl*), and *Dissensus* (*Di*).
  (iv) For $$B\in \{PC, Co, Po, Cl, Di\}$$:
  (A) Answer the Bernoulli trial: *Does the predicted opinion distribution belong to category* $B$?
  (B) If the answer is yes, increase the counter $N_{s,i,j,B}$ by one

(iii) For $$B\in \{PC, Co, Po, Cl, Di\}$$
  (i) Compute the probability
    $$\begin{aligned} {\mathbb {P}}_{i, j, B} = \frac{N_{s,i,j,B} + \frac{1}{2}z^2}{N_e+ z^2} \end{aligned}$$
  (ii) Store the probability ${\mathbb {P}}_{i, j, B}$ in row *i*, column *j* of the table $T_B$.

6. Plot the completed tables $T_{PC}$, $T_{Co}$, $T_{Po}$, $T_{Cl}$, $T_{Di}$ (each with ${\mathcal {I}}$ rows and ${\mathcal {J}}$ columns with values in the interval [0, 1]) using the colour map *CM* to obtain **QOL Tables**. If desired, also plot histograms of the values contained in the tables (the plots will be similar to the ones presented in Fig. 4).
  **Remark:** if in the table plots there are no distinguishable patterns, it is possible to reorder the elements in the ordered families $\{{\mathcal {O}}_i\}_{i=1}^{\mathcal {I}}$ and $\{{\mathcal {P}}_j\}_{j=1}^{\mathcal {J}}$ (which is equivalent to permuting rows and columns of the table) so as to reveal hidden patterns.
7. Since each element in $\{{\mathcal {O}}_i\}_{i=1}^{\mathcal {I}}$ (respectively, $\{{\mathcal {P}}_j\}_{j=1}^{\mathcal {J}}$) is associated with a point in the Agreement Plot (resp. Parameter Plot) by Eq. (2) (resp. (3)), the ordering can be represented by colour coding points in the Agreement Plot (resp. Parameter Plot). This will be useful in the analysis of the **QOL Tables** (resulting in plots such as the ones presented in Fig. 3).

8. To create **QOL Figures**, do the following: For $B\in \{PC, Co, Po, Cl, Di\}$:
  - Initialise an empty Cartesian Plane.
  - For $$i=1, \dots , {\mathcal {I}}$$
  (i) Add all the Bernoulli trial successes of simulations for which the initial opinion distribution belonged to ${\mathcal {O}}_i$. Numerically, this is equivalent to:
    $$\begin{aligned} N_{s,i,\cdot ,B} = \sum _{j=1}^{\mathcal {J}}N_{s,i,j,B} \end{aligned}$$
  (ii) Compute the probability
    $$\begin{aligned} {\mathbb {P}}_{i, \cdot , B} = \frac{N_{s,i,\cdot ,B} + \frac{1}{2}z^2}{{\mathcal {J}}N_e+ z^2} \end{aligned}$$
  (iii) Plot a marker in the Cartesian Plane in coordinates $\big ({\overline{x}}_i, \overline{|x|}_i\big )$. The colour of this marker is given by the value ${\mathbb {P}}_{i, \cdot , B}$ and the colour map *CM*.

(iii) Initialise an empty Cartesian Plane.
(iv) For $$j=1, \dots , {\mathcal {J}}$$
  (i) Add all the Bernoulli trial successes of simulations for which the agent parameter set belonged to ${\mathcal {P}}_j$. Numerically, this is equivalent to:
    $$\begin{aligned} N_{s,\cdot ,j,B} = \sum _{i=1}^{\mathcal {I}}N_{s,i,j,B} \end{aligned}$$
  (ii) Compute the probability
    $$\begin{aligned} {\mathbb {P}}_{\cdot , j, B} = \frac{N_{s,\cdot ,j,B} + \frac{1}{2}z^2}{{\mathcal {I}}N_e+ z^2} \end{aligned}$$
  (iii) Plot a marker in the Cartesian Plane in coordinates $\big ((p_a)_j, (p_b)_j\big )$. The colour of this marker is given by the value ${\mathbb {P}}_{\cdot , j, B}$ and the colour map *CM*.

The results of the previous plots are equivalent to the ones presented in Fig. 5.

In principle, the Agreement Plot and Agent Parameters can be ordered as desired so as to produce **QOL Tables** where patterns are more clearly visible. However, if the ordering is too intricate, the interpretation of **QOL Tables** becomes difficult. This leads to a trade-off between producing easy-to-interpret orderings and **QOL Tables** with clear patterns.

## Results

Following our proposed three-step process, described in detail in the Methods section, we analyse four opinion formation models: the Friedkin-Johnsen (FJ), Classification-based (CB), Backfire Effect and Biased Assimilation (BEBA), and Bounded Confidence (BC) models.

### Agreement plot and parameter plot ordering

Figure 3 shows how the points in the Agreement Plot and in the Parameter Plot can be ordered (differently) for the FJ, CB, BEBA, and BC models.

For the **FJ model**, when the Agreement Plot points are ordered as in Fig. 3a (thus matching the order of rows in the **QOL Tables**), the numbering grows from the corners (0, 0) and (1, 1) to the corner (1, 0). Hence, the first rows of the corresponding **QOL Tables** are approximately associated with opinion distributions that are initially categorised as perfect consensus, in the following rows as consensus, then as clustering, then as dissensus, and eventually as polarisation in the last rows. As for the order of the Parameter Plot points, the first columns correspond to societies with very high susceptibility values, the following columns to societies with a lower susceptibility, while the last columns correspond to societies where every agent has the same value of susceptibility (around 0.3). The order is more strongly affected by the average than by the variance.

For the **CB model**, Fig. 3b shows that the first rows of the **QOL Tables** correspond to societies with very extreme opinions (high $\overline{|x|}$), while the last rows to societies that are almost completely indifferent. The first columns are associated with highly radical societies (high ${\overline{\beta }}$), while the last columns have on average a small radical weight and any proportion of conformist and stubborn weights.

For the **BEBA model**, the order of the Agreement Plot points is shown in Fig. 3c (and is quite similar to the one in Fig. 3a): the first rows in the **QOL Tables** approximately correspond to initial opinion distributions forming perfect consensus, while moving towards the last rows the initial opinion distribution changes from perfect consensus to consensus, dissensus, clustering and, finally, polarisation. On the other hand, the order of the Parameter Plot points is peculiar, and highly depends on both the average and the variance of the agents’ entrenchment. The first columns correspond to low values of entrenchment and as the order increases two groups appear, one with high average entrenchment and the other with intermediate-high average entrenchment and high variance. Therefore, when analysing the **QOL Tables** for the BEBA model, we need to take into account that right columns may correspond to societies where the average entrenchment is very high, or where only *some* agents have a very high entrenchment (this would explain the average of around 4 and high variance).

For the **BC Model**, the Agreement Plot ordering of Fig. 3d means that the first rows in the **QOL Tables** correspond to perfect consensus and move through consensus, clustering, and dissensus until reaching polarisation for the last rows. The agent parameter ordering shows a significant dependence on the confidence radius variance: the first columns correspond to high mean radius with low variance and the last columns to intermediate-low mean confidence radius and high variance. Hence, in the societies represented in the left columns in the **QOL Tables**, most agents have the same confidence radius, around 2, and in societies to the right some agents have a high and others have a low confidence radius.

### Qualitative outcome likelihood tables

Figure 4 presents the **QOL Tables** for the FJ, CB, BEBA, and BC models. Here we briefly discuss the results achieved for each of the models and highlight the peculiarities of their behaviour.

#### FJ model

Figure 4a shows that perfect consensus is an almost certain outcome for the first rows. In fact, since the opinion distributions in these rows are initially located around the corners (0, 0) and (1, 1), they start as perfect consensus and, given that the FJ model has no mechanism to diversify opinions, the opinion distributions remain categorised as perfect consensus. Perfect consensus is also almost sure for cells in the left columns, corresponding to societies with very high susceptibility. In fact, if all agents are highly susceptible, they reach perfect consensus, because in these cases the FJ model boils down to the French-DeGroot model.

Figure 4a also shows that polarisation can be expected only when agents start from an already polarised opinion distribution and the susceptibility is very low and uniform (see the orange bottom right corner in the third table): the agents remain with their initial opinion, which was polarised from the start. Also other opinion distribution categories have regions in which they are more likely, but with a less intuitive interpretation.

The histograms in Fig. 4a provide additional information that is independent of the row and column order. The only category which can be almost guaranteed as an outcome is perfect consensus: only in this case, the right bin (in the neighbourhood of probability 1) has a significant height. Also, the least likely outcome is polarisation: the corresponding histogram has the largest left bin (in the neighbourhood of probability 0). The consensus histogram has an almost uniform probability distribution, meaning that the outcome probability highly depends on missing information.

#### CB model

Only perfect consensus and consensus are likely to occur in well-defined regions in the **QOL Tables** in Fig. 4b, suggesting that this model’s behaviour is highly intricate. Other Agreement Plot and Parameter Plot orders may create better defined regions, but would not follow an easy-to-interpret pattern and, as such, they would not provide interpretable information about the model behaviour.

The histograms show that, except for dissensus, all qualitative outcomes have a non-negligible probability of being achieved. For every category, a significant number of cells has a probability different from zero or one. Interestingly, perfect consensus and consensus are likely outcomes in opposite scenarios: perfect consensus occurs in the left columns, corresponding to highly radical societies, while consensus can be found in societies with low radicalism. This happens because radicalism moves opinions to extremes, which often results in perfect consensus (the only other outcome produced by radicalism is polarisation, but it requires the initial opinions to have almost null bias, which is rarely the case, hence the most likely outcome is perfect consensus, as one extreme will have significantly more agents that the other), while consensus requires a clear but not extreme tendency of all agents towards similar opinions, which is more likely achieved by high conformist traits. Clustering is partially present in the left side of the last rows, corresponding to significantly radical societies starting from almost indifferent opinions. In fact, individuals with radical traits may move from indifference towards a stronger opinion, but not to such an extent to produce perfect consensus: they may end up forming two or three not very distant subgroups that correspond to clustering.

Comparing Fig. 4a and b reveals that perfect consensus and consensus, which have a high probability in adjacent cells for the FJ model, have a high probability in opposite cells for the CB model.

#### BEBA model

Figure 4c shows that the BEBA model has a strong tendency to transform opinion distributions into either perfect consensus or polarisation. In particular, perfect consensus is by far the most likely outcome, which occurs almost always; in the rare cases in which perfect consensus is not achieved, then the outcome is polarisation. This can be somewhat unexpected, as backfire effect and biased assimilation are generally associated with polarisation. However, according to our definition, polarisation requires the existence of two ‘distant enough’ subgroups *having ‘comparable’ size* (The formal definition of ‘distant enough’ and ‘comparable’ group size depends on the interpretation and code implementation; the quantitative metrics adopted in the manuscript are explained in detail in the Supplementary Information file, where the algorithm to sort opinion distributions is described. In particular, in our case, ‘distant enough’ means that the difference between opinions is at least 0.6, while ‘comparable size’ means that the difference between the numbers of individuals in the two groups is small enough, at most amounting to $38\%$ of the population in the most extreme case: in fact, none of the two groups includes more than $50\%$ of the population or less than $12\%$ of the population, but the two groups together include more than $50\%$ of the population.). If this last condition is not met, e.g. because one group has significantly more individuals than the other, then the opinion distribution is categorised as perfect consensus. Although the BEBA model promotes the existence of these two separate subgroups, it includes no intrinsic mechanism to equalise the number of individuals in each one and, since random initial assignations most likely produce unequal subgroups, most of them are categorised as perfect consensus.

The conditions for which the BEBA model most likely produces polarisation can be inferred from Fig. 4c: polarisation mostly occurs in the lower right corner, which corresponds to initial opinions near the (1, 0) point in the Agreement Plot (associated with a polarised distribution), and agent parameters with either high average entrenchment or intermediate-high average entrenchment and high variance. Then, if the population is already polarised, high entrenchment ensures that the two separate subgroups forming the initial polarisation distribution are maintained.

#### BC model

The **QOL Tables** in Fig. 4d show that the Bounded Confidence model predominantly produces perfect consensus and that the second most likely outcome is clustering. Perfect consensus is almost guaranteed for the left part of the **QOL Tables**, that is, approximately when the mean confidence radius is above 1. This makes sense: with such a high mean confidence radius, even with high variance, several agents will be influenced by almost all the other agents and cause global convergence to a single opinion, producing perfect consensus.

The **QOL Tables** show that clustering is almost guaranteed for the bottom right corner, that is, when the initial opinions are near the point (1, 0) (most likely polarised), and the confidence radius has an intermediate-low mean (around 0.5) and high variance (around 0.8). This happens because, when the initial opinions start from a polarised condition, the agents with intermediate confidence radius move towards the agents with low confidence radius, which remain in place, as they are influenced by very few agents. This behaviour has the potential of moving agents away from the two initial polarised groups and thus lead to clustering.

From Fig. 4d it is also clear that the other qualitative outcomes are extremely unlikely, because, in order to exist, they would need to start from very specific initial opinions, and the initial opinions and agent parameters would require a very specific configuration, which is not likely to happen at random.

### Qualitative outcome likelihood figures

Figure 5 presents the **QOL Figures** for the FJ, CB, BEBA, and BC models.

#### FJ model

Figure 5a confirms that perfect consensus is the only outcome that occurs with very high probability for some regions of the Agreement Plot and the Parameter Plot. Looking at the Agreement Plot, this happens when the average of the opinions and of their absolute values is in the the neighbourhood of corners (0, 0) or (1, 1): opinions starting from perfect consensus are very likely to remain in that category, since the FJ model includes no mechanism to move opinions away from each other. Also, looking at the Parameter Plot, perfect consensus is very likely when the average susceptibility is high: in this case, the model behaves as the French-DeGroot model, which is known to lead to perfect consensus.

In addition to these clear trends, analysing the other plots of Fig. 5a reveals interesting observations. For both the Agreement Plot and the Parameter Plot, the consensus probability is significant (around 50%) for a wide variety of initial opinions and agent parameters, as shown by the vast purple area. Conversely, the probabilities of obtaining other opinion categories are low. Polarisation can only occur in the FJ model when the opinions are initially polarised and the susceptibility is low. Similarly, clustering can only happen when the opinions start either in clusters or dissensus and the agent parameters are ‘uniformly’ allocated, so that in the proximity of different opinions some agents have a low susceptibility (and thus keep their own opinion) while others have high susceptibility (and thus move towards the agents with low susceptibility). Although this combination of circumstances is unlikely, it is still noticeable in the plots corresponding to clustering, especially in the purple colour of Parameter Plot points associated with high variance and low average of the susceptibility.

#### CB model

Unlike the **QOL Tables** in Fig. 4b, the plots in Fig. 5b show clear regions in the Agreement Plot and Parameter Plot where specific qualitative outcomes are likely to be achieved. Perfect consensus, polarisation and clustering have clear regions in the Agreement Plot associated with significant probability: the neighbourhood of points (1, 1), (1, 0), and (0, 0) for perfect consensus, polarisation, and clustering respectively. For perfect consensus and polarisation, those opinions already belong to the desired qualitative category, therefore, if they do not change much, their qualitative category will not change. The possible explanation for the clustering case was previously discussed.

In the Parameter Plot, perfect consensus, consensus, polarisation, and clustering have regions associated with significant probability, which seem to depend mostly on the average radical weight. From minimum to maximum radical weight, the order in which the qualitative outcomes are more likely is consensus, clustering, polarisation, and perfect consensus. In fact, the existence of these categories requires a degree of intermediate and not extreme opinions: consensus needs multiple similar but not identical opinions, clustering needs two or more subgroups with distinguishable opinions, polarisation needs two subgroups with a comparable number of agents having distant opinions, and perfect consensus needs most agents having very similar, and possibly extreme, opinions. The average radical weight directly relates to how extreme opinions are in the population.

#### BEBA model

Figure 5c (row 2, column 1) shows that perfect consensus is by far the most likely outcome, even in the presence of high entrenchment. This further corroborates the conclusion, drawn from Fig. 4c, that high entrenchment does not necessarily lead to polarisation. The Parameter Plots in Fig. 5c show that, when all initial opinions are considered, only intermediate-low average values of entrenchment with high variance have a significant probability of producing opinions that are not categorised as perfect consensus.

#### BC model

Figure 5d provides more specific information about the conditions for which perfect consensus is not the almost sure outcome. This happens when the variance is maximal and the mean confidence radius is below 1 (see the purple area in row 2, column 1 of Fig. 5d). In that region, there is also a significant probability of obtaining clustering, and when the mean confidence radius is below 0.5 there is a slight probability of obtaining polarisation. This happens, however, not because opinion distributions from different categories evolve towards polarisation, but because polarised opinions remain polarised.

### Global model probability outcomes

Table 1 reports the outcome probabilities when no information at all is known about both the initial opinion distribution and the agent parameters, and therefore shows the approximate probabilities that an opinion formation model yields each of the five possible outcomes. Again the FJ, CB, BEBA, and BC models are considered. Interestingly, the FJ model is the only for which perfect consensus is not the most likely outcome, despite being one of the two models without a mechanism to disperse opinions (the other one is the BC model): in fact, the susceptibility trait in this model allows opinions to move close, but prevents them from converging to the exact same opinion. Both the FJ and the CB models show a remarkable ability to yield all the different qualitative behaviours, although polarisation is relatively unlikely for the FJ model and dissensus is relatively unlikely for the CB model. Conversely, the BC and BEBA models are strongly biased towards perfect consensus. It can also be observed that, for the BEBA and BC models, the second most likely outcome (polarisation for the BEBA model, clustering for the BC model) has approximately the same probability.

**Table 1:**
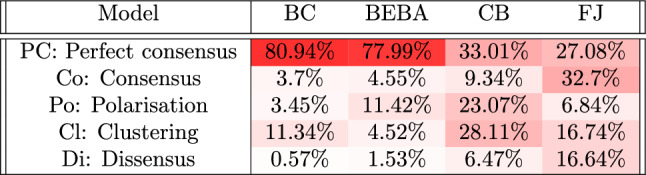
Probability of obtaining an opinion distribution of the corresponding qualitative category (Perfect consensus, Consensus, Polarisation, Clustering, Dissensus) for each opinion formation model (Friedkin-Johnsen, Classification-based, Backfire Effect and Biased Assimilation, and Bounded Confidence) after 50 time steps. The cell colour saturation is proportional to the reported probability. The number of samples used to compute this probability, for each model is: 1198860000, 1154340000, 1014420000, and 1179780000 respectively (10,000 times the number of cells in the corresponding **QOL Tables**).

## Discussion

We have proposed a novel numerical simulation-based analysis technique for agent-based opinion formation models. Starting from incomplete information on the considered population (initial opinion distribution, agent parameters, underlying digraph), we can approximate the probability that the predicted opinion distribution belongs to a qualitative category (perfect consensus, consensus, polarisation, clustering, or dissensus). The systematic application of this approximation can be used to construct Qualitative Outcome Likelihood Tables and Figures, which reveal intrinsic model properties and behaviour patterns. We have applied the proposed technique to study four agent-based opinion formation models: Friedkin-Johnsen (FJ), Classification-based (CB), Backfire Effect and Biased Assimilation (BEBA), and Bounded Confidence (BC).

For the FJ model, our results suggest that the average population susceptibility ${\overline{\lambda }}$ is a good indicator of the predicted opinion distribution category. Moving from complete average susceptibility ${\overline{\lambda }} = 1$ to null average susceptibility ${\overline{\lambda }} = 0$, the most likely qualitative outcomes are perfect consensus, consensus, dissensus, clustering, and polarisation. Polarisation is the least likely outcome and only occurs when the initial condition is already at polarisation: in fact, the FJ model includes no mechanism to move opinions apart. For the same reason, the only outcome that occurs almost certainly in some conditions (when the initial opinions are either indifferent, near 0, or extreme, near 1 or $-1$) is perfect consensus.

However, including a mechanism that drives opinions apart is not guaranteed to increase the likelihood of polarisation, as shown by the results with the BEBA model. Although the Backfire Effect does move opinions in opposite directions, polarisation is a very unlikely outcome. On the contrary, compared to the FJ and CB models, the BEBA model predominantly produces perfect consensus. In fact, polarisation not only requires opinions to be significantly distant, but also requires the formation of two distant groups including a very similar number of agents, which rarely happens when the initial opinions are random. If the initial opinions have a significant bias, then moving opinions to opposite extremes simply results in a greater majority of agents having one extreme opinion, leading to perfect consensus.

Like the FJ model, the CB model has a significant potential of producing opinion distributions that do not form perfect consensus. Our results for this model suggest that it has a particularly complex behaviour, since the **QOL Tables** do not display many clearly distinguishable regions associated with qualitative behaviours. However, the average radical trait is shown to have a significant effect on the model outcome.

The analysis of the BC model showed that, although this model is widely related to clustering behaviour, this outcome is not the most likely, and in fact occurs in very particular circumstances, when the initial opinions are polarised or evenly distributed, and the distribution of agent’s confidence radii has mean around 0.5 and variance around 0.8. In general, for this model, a uniformly distributed agent parameter set creates more variety in the predicted opinions, because heterogeneous confidence radii result in a network that is not necessarily strongly connected and therefore allows agents to have different opinions. On the other hand, if all the confidence radii are similar, then the agents are either all isolated or strongly connected.

The Agent Parameter ordering plots in Fig. 3 suggest that the mean of the agent parameters is the most significant global parameter in the qualitative description of the model behaviour. On the one hand, this is not surprising, as the mean of a set of numbers is one of its most fundamental properties. In this context, the mean parameter characterises the average agent and is thus a good measure of how the population behaves collectively. For instance, for the FJ model, knowing that the average agent has a high susceptibility is more relevant than knowing that all agents have the same—unknown—susceptibility. On the other hand, it is remarkable that a simple indicator like the mean can capture the overall behaviour of such complex systems. In fact, this is not always the case: there could be models where the Agent Parameter ordering can vary significantly along both axes. The BEBA and BC plots in Fig. 3 suggest how these orderings could look like.

It is worth stressing that our analysis does not require any assumption on the convergence to an equilibrium. Our proposed **QOL Tables** and **QOL Figures** provide a static snapshot of the evolution of social systems, suitably sampling the parameter space and the space of possible initial conditions, after a fixed number of time steps. If, for the considered model and the chosen simulation horizon, an equilibrium has been reached at the end of the simulation, then **QOL Tables** and **QOL Figures** capture the asymptotic behaviour of the system. On the other hand, our probabilistic analysis approach could also be adopted to study non-stationary asymptotic behaviours, which have been observed in some models of opinion dynamics^47,48^. In fact, the analysis of a sequence of **QOL Tables** and **QOL Figures** corresponding to different simulation horizons can provide further insight into the overall temporal evolution and properties of a given model, revealing transient or periodic behaviours.

If **QOL Tables** and **QOL Figures** are used to analyse the asymptotic model behaviour, all the simulations need to have reached equilibrium. Heuristic approaches can be adopted to properly choose the simulation horizon, so as to guarantee that the results of such an analysis are sound and interpretable. A sequence of **QOL Tables** and **QOL Figures** for increasing time horizons can be compared and, once no change is detected, all systems can be assumed to have reached equilibrium. As an alternative, we can adopt a procedure similar to that used to construct **QOL Tables** and **QOL Figures**, with the Bernoulli trial being the answer to the question: *Has the system reached equilibrium?*

The proposed technique is part of a recent trend of simulation-based analysis methodologies and frameworks used to study and compare opinion formation models that, because they include various social and psychological traits, are too complex to be thoroughly assessed analytically. As such, the proposed *QOL analysis* can be used in conjunction with other recently proposed approaches, such as the Transition Tables^38^, the Agreement Plot^39^, the Global Unifying Frame^41^, and the distributional measures of *Bias*, *Diversity*, and *Fragmentation*^13^.

Importantly, our proposed *QOL analysis* technique accounts for incomplete information to reflect reality: for social systems, the model parameters and the initial opinions are extremely challenging or even impossible to estimate, and this crucial aspect cannot be overlooked by assuming exact initial conditions and parameter values.

Another advantage is that the systematic application of the proposed technique allows for a comprehensive exploration of the model capabilities from a wide range of initial conditions and model parameters: different initial opinions (not only uniformly random, which is the case in most simulation results), agent parameters (that are frequently assumed to be the same for all agents), and underlying digraphs (not only regular graphs). This, in turn, offers a clearer and broader picture of the opinion distributions that a model can produce.

On the other hand, this thorough analysis requires a significant computational burden: many simulations need to be performed to obtain meaningful results. For instance, creating the **QOL Tables** in Fig. 4a has required the simulation of approximately $(318)(371)(10,000) = 1.1798 \cdot 10^{9}$ different social systems. Even with code optimisation and multi-core computers, this is a considerable effort. Furthermore, the results only provide qualitative information on the system outcome.

In particular, the amount of time $T_\text {sim}$ needed to produce the **QOL Tables** depends on the model, the simulation horizon, the density of samples in the parameter and initial condition space, and the number of events (number of Bernoulli trials). The product of the simulation horizon *K* and the single step calculation time $T_\text {step}$ approximates the time a single model evolution requires, while the product of $N_e|\{{\mathcal {O}}_i\}||\{{\mathcal {P}}_j\}|$ is the total number of simulations (with |*X*| denoting the cardinality of set *X*). Since all the simulations are independent, the analysis is easily parallelizeable: if $N_e|\{{\mathcal {O}}_i\}||\{{\mathcal {P}}_j\}|$ cores are available, then $T_\text {sim}\approx KT_\text {step}$; on the other hand, if a single core is available, then $T_\text {sim}\approx N_e|\{{\mathcal {O}}_i\}||\{{\mathcal {P}}_j\}|KT_\text {step}$. In general, if $N_\text {cores}$ cores are used to compute the different simulations, then $T_\text {sim}\approx \lceil N_e|\{{\mathcal {O}}_i\}||\{{\mathcal {P}}_j\}|/N_\text {cores}\rceil KT_\text {step}$, where $\lceil \cdot \rceil$ is the ceiling operator. The single step calculation time, $T_\text {step}$, depends in turn on the model, the number of agents, the hardware, and the code implementation. However, in practical applications it will be at most of the order of milliseconds (in a 2.3 GHz Intel Core i5 processor, the value of $T_\text {step}$ is about 0.343 ms, 0.377 ms, 0.182 ms, and 0.015 ms for the BC, BEBA, CB, and FJ models respectively, implemented in Matlab). In a single core case, with the parameters used for the FJ model ($K=50$, $N_e=10,000$, $|\{{\mathcal {O}}_i\}|=318$, and $|\{{\mathcal {P}}_j\}|=371$) a total of $5.9\cdot 10^{10}$ model time-steps are required, which take approximately $8.64\cdot 10^5$ seconds, or about 10 days. The classification algorithm to decide the Bernoulli trial needs to be executed $N_e|\{{\mathcal {O}}_i\}||\{{\mathcal {P}}_j\}|$ times, which can add significant time to the overall computations, so that, even with multiple cores available, the simulations can take several days. The dependence of $T_\text {step}$ on the number of agents varies with the model. For models like the weighted-median model^11^, $T_\text {step}$ is independent of the number of agents. For models like the BC model or the CB model, the number of operations grows polynomially with the number of agents. In our simulations, we considered 100 agents, a number that is big enough for the system to exhibit complex behaviours, but small enough to make the simulation time reasonable. Although the algorithm is easily parallelizeable, since the simulations are independent, the computational burden is a limiting factor when exploring more dense parameter space and initial condition space, and longer simulation horizons.

The choice of simulation horizon *K*, number of trials $N_e$, and size of the families $\{{\mathcal {O}}_i\}$ and $\{{\mathcal {P}}_j\}$ depends on the analysis requirements and available resources. The simulation horizon can be chosen by fixing the time interval to be investigated and estimating the number of opinion changes the agents can have in this time interval. For our case, we considered a time interval of 5 years and estimated 10 opinion changes per year (as done in^38^ to align with the World Values Survey timeline), resulting in $K=50$. The number of trials $N_e$ depends on the desired confidence. As seen in Eq. (1), $\delta \rightarrow 0$ as $N_e\rightarrow \infty$. In our case, we chose $N_e=10,000$ so that all the calculated probabilities had an uncertainty of at most 0.01 (see Fig. 1 in the Supplementary Information). Equation (1) can thus be used when determining the needed number of trials. The reasonable size of the families $\{{\mathcal {O}}_i\}$ and $\{{\mathcal {P}}_j\}$ depends on the purpose for which the **QOL Tables** are constructed. Since we plotted the **QOL Tables** for visual inspection, the appropriate size of $\{{\mathcal {O}}_i\}$ and $\{{\mathcal {P}}_j\}$ was determined by the desired image resolution. On print, the plots of the **QOL Tables** would be at about one inch per side, and 300 pixels/inch is a standard high-quality printing resolution: hence, we chose the number of samples so that tables had around 300 rows (size of $\{{\mathcal {O}}_i\}$) and columns (size of $\{{\mathcal {P}}_j\}$). Given the limited resolution that the human eye can perceive, taking more samples would provide comparable results, while requiring more resources. Of course, the size can be freely chosen when implementing the methodology so as to comply with different requirements.

Another factor that could affect the size of $\{{\mathcal {P}}_j\}$ is the parameter space of the model. The parameter space is bounded in the FJ model (susceptibility lies in the interval [0, 1]), in the BC model (the confidence radius lies in the interval [0, 2]) and in the CB model (the possible agent traits are bounded in the simplex $\alpha \ge 0$, $\beta \ge 0$, $\gamma \ge 0$, and $\alpha +\beta +\gamma =1$). Conversely, for the BEBA model the entrenchment can be any positive value ($\rho >0$). When the parameter space is bounded, taking a family $\{{\mathcal {P}}_j\}$ that covers all the parameter space is equivalent to carrying out a sensitivity analysis, exploring the range of all possible parameters; therefore, our approach lends itself to be used as a tool for sensitivity analysis, showing the impact of choosing different parameter values. On the other hand, when the parameter space is unbounded, a preliminary sensitivity analysis is needed to determine the portion of the parameter space that $\{{\mathcal {P}}_j\}$ will cover. In the case of the BEBA model, we found out that choosing an entrenchment value higher than 7 yields extreme model behaviours that lead to complete polarisation almost immediately, and hence we considered entrenchment values bounded as $0<\rho <7$.

The proposed technique is very versatile and can be used to study any agent-based opinion formation model, as long as the opinion of every agent is a bounded real number. Additionally, the Bernoulli trial can be modified to be anything the study requires. So, for instance, instead of assigning the predicted opinion distribution to one of the five categories, the Bernoulli trial could be asking whether the predicted opinion distribution meets certain desired criteria (such as being in a given subinterval). Another possibility is to use the probabilistic analysis to study the effect of the graph topology on the model evolution. Here, we have considered digraphs with a Small-World network topology because they possess high clustering coefficient and low diameter, characteristics that have been observed in real-life societies^49,50^. However, by simply changing the set ${\mathcal {N}}$ to contain digraphs with e.g. Scale-Free or Random topologies, and comparing the resulting **QOL Tables** and **QOL Figures**, the overall effect of the network topology on the model behaviour can be studied via our proposed methodology.

The predictions offered by recent agent-based opinion formation models explain some opinion evolutions seen in real life, but they still do not account for other ones. To gain further insight into the complex mechanisms behind opinion formation, increasingly more sophisticated and intricate models are likely to be proposed, and their analysis will require thorough numerical techniques such as the one we proposed in this paper.

### Supplementary Information

Supplementary Information.

## Data Availability

All our code is available at https://giuliagiordano.dii.unitn.it/docs/papers/PAcode.zip.
