# Supplementary material for: Probabilistic analysis of agent-based opinion formation models
Source: Sci Rep. 2023 Nov 17;13:20152. doi: 10.1038/s41598-023-46789-3 (PMC10656423; doi:10.1038/s41598-023-46789-3)
Supplement: Supplementary file 1 — Supplementary Information. [file 41598_2023_46789_MOESM1_ESM.pdf]

Probabilistic Analysis of Agent-Based Opinion Formation Models — Supplementary Information

Carlos Andres Devia[1] and Giulia Giordano[1,2,*]

[1]Delft Center for Systems and Control, Delft University of Technology, 2628 CD Delft, The Netherlands

[2]Department of Industrial Engineering, University of Trento, 38123 Trento, Italy

[*]giulia.giordano@unitn.it

Abstract

This Supplementary Information for the paper *Probabilistic Analysis of Agent-Based Opinion Formation Models* provides: a detailed description of the sorting algorithm; a detailed description of the simulation procedure used to produce the results presented in the paper; a quantification of the maximum uncertainty radius for the probability assessment; the procedure for the construction of the sets of initial opinion distributions, agent parameter sets and underlying digraph topologies; a step-by-step guide to use the provided code; and high resolution figures.

Contents

1 Algorithm to Sort Opinion Distributions **1**

2 Probabilistic analysis: the procedure **2**

3 Uncertainty radius for the probability assessment **2**

4 Construction of the sets $\mathcal{O}$, $\mathcal{P}$, $\mathcal{N}$ **2**

4.1 Set of initial opinion distributions 3

4.2 Set of agent parameters sets 3

4.3 Set of underlying digraphs 4

5 Network Metrics **6**

6 Code usage **7**

6.1 Steps to reproduce the results 7

6.2 Datasets 7

References **8**

A High Resolution Figures **9**

1 Algorithm to Sort Opinion Distributions

An opinion distribution can be visualised as a histogram with M bins, which can then be sorted (namely, classified) so as to determine to which qualitative category the opinion distribution belongs. This process is performed by our proposed *Histogram-based Sorting Algorithm*:

1. Input the positive integers M, $B < M$, $K \leq M - 2$ and $\Upsilon \leq M - 2$, and the thresholds T_1, T_2, and T_3, with $0 < T_2 < T_3 < 50 \leq T_1 < 100$.
2. Partition the $[-1, 1]$ interval in M bins of equal width.
3. Count how many opinions fall in each bin, and denote by $H(k)$ the number of opinions in bin k $(1 \leq k \leq M)$.
4. Normalise the bin counts so they add up to 100, and denote the normalised bin counts by $\tilde{H}(k)$.
5. Classify each bin as *green*, *blue*, or *red*: bin k is green if $\tilde{H}(k) > T_1$; blue if $\tilde{H}(k) < T_2$; red otherwise.
6. Compute the *number of groups*; a group is formed by consecutive green or red bins. For each group, compute the *number of bins*, and the *normalised group count*, which is the sum of all the normalised bin counts of the bins belonging to the group.
7. Sort the histogram according to the following criteria:

- *perfect consensus* if there is a green bin;
- *consensus* if there is one group, with at most B bins, and with normalised group count larger than 50;
- *polarisation* if there are two groups, each with at most B bins, with at least K bins in between, whose normalised group counts add up to more than 50;
- *clustering* if there are two or more groups, each with at most B bins, whose normalised group counts add up to more than 50;
- *dissensus* otherwise.

8. The only exception to the previous criteria is if the histogram has only two non-empty bins with at least Υ empty bins in between and each of these two non-empty bins has a normalised group count larger than T_3. Then the opinion distribution is also classified as polarisation.

The algorithm described above, which we used in this paper, is a modification from the algorithm presented in [1]: the novelty here is that we have introduced the exception in item 8.

Such an exception has been added because, when the histogram has only two non-empty bins, it will always be classified as perfect consensus, unless both bins have the exact same height (i.e. 50). However, intuitively, if there are only two non-empty bins at a significant distance, of comparable height, the histogram should be classified as polarisation.

The parameters used in this paper to run the algorithm are: $M = 10$, $B = 3$, $K = 4$, $\Upsilon = 6$, and the thresholds $T_1 = 50$, $T_2 = 12$, $T_3 = 40$.

2 Probabilistic analysis: the procedure

We recall our proposed three-step process to compute the probability $\mathbb{P}_B(\mathcal{O}, \mathcal{P}, \mathcal{N}, K)$ that, if the initial opinion distribution belongs to the set $\mathcal{O}$, the agent parameter set belongs to the set $\mathcal{P}$, and the underlying digraph belongs to the set $\mathcal{N}$, the final predicted opinion distribution after K time steps is categorised as $B \in \{PC, Co, Po, Cl, Di\}$:

1. Create three algorithms that randomly sample from the sets $\mathcal{O}$, $\mathcal{P}$, and $\mathcal{N}$ uniformly.
2. For a number of events N_e, do the following:
   (a) Randomly select uniformly an element from $\mathcal{O}$, $\mathcal{P}$, and $\mathcal{N}$,
   (b) evolve the system with the selected initial opinion distribution, agent parameter set, and underlying digraph according to the opinion model for the designated time steps,
   (c) categorise the final opinion distribution according to the algorithm described in the previous section (Algorithm to Sort Opinion Distributions): if the resulting category is the desired one, then the event is labelled as a success.
3. The previous sequence of N_e events has the structure of a Bernoulli process, where the random variable is a success if the final opinion distribution belongs to the chosen category. Hence, the number of events N_e and successes N_s can be used to approximate the probability that the final opinion distribution actually belongs to the desired category, as per Equation (1) in the main paper, which relies on the Wilson score interval [2]:

$$\mathbb{P}_B(\mathcal{O}, \mathcal{P}, \mathcal{N}, K) \in [P - \delta, P + \delta] \quad \text{with probability } 0.95, \tag{1}$$

$$\text{given } P = \frac{N_s + \frac{1}{2}z^2}{N_e + z^2}, \quad \delta = \frac{z}{N_e + z^2}\sqrt{\frac{(N_e - N_s)N_s}{N_e} + \frac{z^2}{4}}, \tag{2}$$

where $z = 1.96$ is the z-value for *95% confidence level*, while N_e and N_s are respectively the number of events and of successes of the considered Bernoulli process.

3 Uncertainty radius for the probability assessment

Figure S1 shows that, since for all our investigations the number of sampled events is $N_e = 10000$, for any possible number of successes $N_s \in \{0, 1, \dots, N_e\}$ the uncertainty δ with which we evaluate the sought probability is less than 0.01. Hence, for all the results we show, $|\mathbb{P}_B - P| < 0.01$ with 95% probability, where P is as defined in the above Equation (1).

4 Construction of the sets $\mathcal{O}$, $\mathcal{P}$, $\mathcal{N}$

Although the procedure in Section 2 looks conceptually simple, step 1 is particularly challenging. Creating an algorithm that is guaranteed to uniformly sample elements from the sets $\mathcal{O}$, $\mathcal{P}$, and $\mathcal{N}$ can be very complicated, depending on the formal definition of these sets. With this in mind, we now explain in detail how these sets are chosen for our numerical implementation of the proposed methodology, considering $N = 100$ agents.

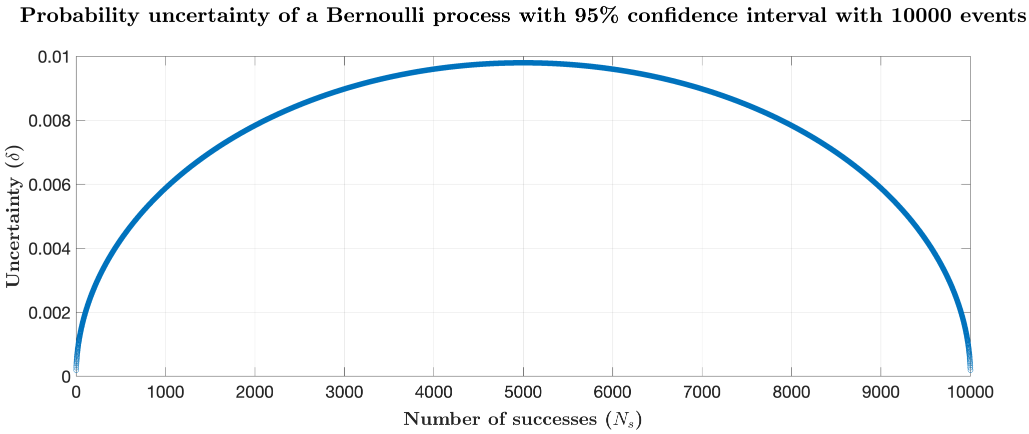

Figure S1: Uncertainty δ affecting the approximate probability of the Bernoulli process according to Equations (1)-(2) when the number of events is $N_e = 10000$. Regardless of the number N_s of successes, the uncertainty δ is always lower than 0.01.

4.1 Set of initial opinion distributions

The set of opinions an agent can have is $O = \{-1, -1+\Delta, -1+2\Delta, \ldots, 1-2\Delta, 1-\Delta, 1\}$, where $\Delta = 2/14$. Numerical quantisation of the interval $[-1, 1]$ entails that each agent can only choose among 15 possible opinions: $O = \{X_k\}_{k=1}^{15}$. Hence, the set of possible initial opinion distributions is constrained as

$$\mathcal{X} = \left\{ x = \{x_i\}_{i=1}^{100} \colon\ x_i \in O \text{ for } i = 1, \ldots, 100 \text{ and } \left|\left\{\tilde{x} \in x \colon \tilde{x} = X_k\right\}\right| \in \{0, 10, 20, \ldots, 100\} \text{ for } k = 1, \ldots, 15 \right\}. \quad (3)$$

Therefore, besides the possible opinions an agent can have being discrete (the 15 options in the set O), the number of agents in the opinion distribution x that have each of the possible opinions in O needs to be a multiple of 10. These constraints are needed to ensure that the set of possible opinion distributions $\mathcal{X}$ has a manageable size. In fact, the original set of possible opinion distributions $[-1, 1]^{100}$ has an infinite number of elements, while the set $\mathcal{X}$ in Equation (3) contains "only" 1961256 elements.

Following the intuitive interpretation of the points in the Agreement Plot, we assume to have limited information on the initial opinion distributions which consists of the average of the opinions and of the average of the opinion absolute values. Representing every element in $\mathcal{X}$ by its average and the average of its absolute values produces the Agreement Plot representing the set $\mathcal{X}$ in the Cartesian Plane, which is shown in Figure S2a.

If the average $\overline{x}$ and the average of the absolute values $\overline{|x|}$ of the initial opinion distribution x are only approximately known with uncertainty radius ϵ_o, then the collection of initial opinion distributions that takes into account this uncertainty is

$$\mathcal{O}(\overline{x}, \overline{|x|}, \epsilon_o) = \left\{ \xi \in \mathcal{X} \colon \sqrt{\left(\overline{x} - \overline{\xi}\right)^2 + \left(\overline{|x|} - \overline{|\xi|}\right)^2} \leq \epsilon_o \right\}. \quad (4)$$

The collection defined in Equation (4) is the one used for the QOL analysis. Figure S2b shows an example of how a collection of this form looks like in the Agreement Plot.

4.2 Set of agent parameters sets

The definition of this set depends on the model: each model has peculiar agent parameters that capture psychological and sociological traits. For now we consider the Friedkin-Johnsen model, where each agent is characterised by a single parameter $\lambda_i \in [0, 1]$, denoting its susceptibility. Again, instead of considering all the possible sets of agent parameters, we consider the constrained collection

$$\mathcal{Y} = \left\{ \lambda = \{\lambda_i\}_{i=1}^{100} \colon\ \lambda_i \in \Lambda \text{ for } i = 1, \ldots, 100 \text{ and } \left|\left\{\tilde{\lambda} \in \lambda \colon \tilde{\lambda} = \Lambda_k\right\}\right| \in \{0, 10, 20, \ldots, 100\} \text{ for } k = 1, \ldots, 15 \right\}, \quad (5)$$

where $\Lambda = \{\Lambda_k\}_{k=1}^{15}$ and $\Lambda_k = (k-1)/14$. The set $\mathcal{Y}$ contains "only" 1961256 elements (instead of infinitely many). This time, the relevant properties of the agent parameter set are the average and the variance (since the susceptibility

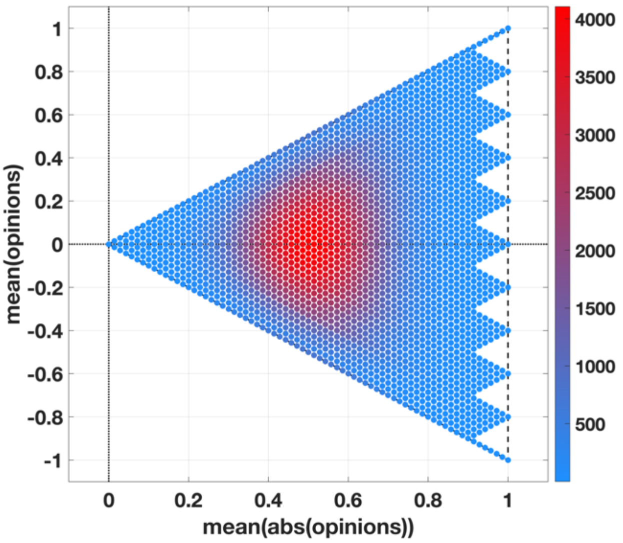

(a) Agreement Plot of the opinion distributions in the set $\mathcal{X}$ as defined by Equation 3. The colour of each dot represents the number of elements in $\mathcal{X}$ that have the same average and average of the absolute values.

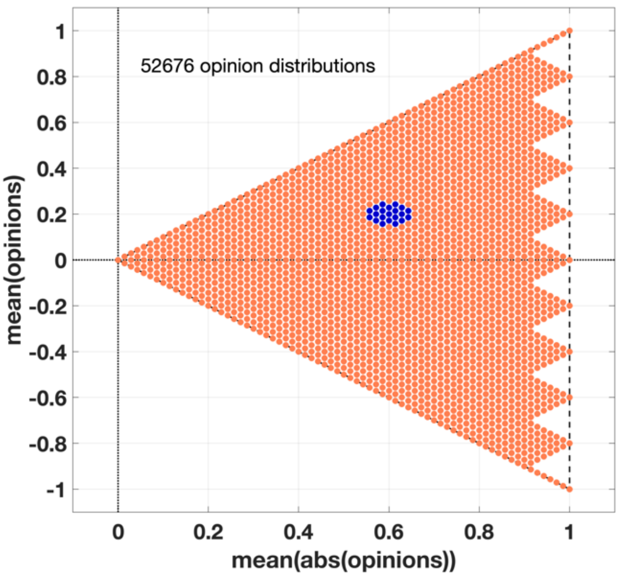

(b) The points highlighted in blue correspond to the set $\mathcal{O}(0.2, 0.6, 0.05)$ according to Equation (4).

Figure S2: Agreement Plot of the opinion distributions in the collection $\mathcal{X}$ according to Equation (3). The vertical saw-tooth pattern is due to the construction of $\mathcal{X}$, in particular due to the restricted (quantised) values that the opinions can take and due to the requirement that each of the possible opinion values must be shared by a number of agents that is a multiple of 10.

is always non-negative, the average of its absolute value is the same as its average). Representing every element in $\mathcal{Y}$ by its average and variance yields the Parameter Plot shown in Figure S3a.

If the average $\overline{\lambda}$ and the variance $\sigma(\lambda)$ of the agent parameters (in the FJ case, susceptibilities λ) are only approximately known, with uncertainty radius ϵ_p, then the collection of agent parameter sets that represents this uncertainty is

$$\mathcal{P}\big(\overline{\lambda}, \sigma(\lambda), \epsilon_p\big) = \left\{ \pi \in \mathcal{Y} \colon \sqrt{\big(\overline{\lambda} - \overline{\pi}\big)^2 + \big(\sigma(\lambda) - \sigma(\pi)\big)^2} \leq \epsilon_p \right\} \tag{6}$$

The collection defined in Equation (6) is the one used for the QOL analysis. Figure S3b shows an example of how a collection of this form looks like in the Parameter Plot.

4.3 Set of underlying digraphs

The set of all possible underlying digraphs also depends on the model, for instance the Friedkin-Johnsen model is associated with the set of all $N \times N$ row-stochastic weighted adjacency matrices, whereas the digraphs in the Altafini model have adjacency matrices belonging to the set $\{-1, 0, 1\}^{N \times N}$, since in that model the digraphs are signed but unweighted. Call $\mathcal{Z}$ the set of adjacency matrices for all possible underlying digraphs admitted by the model, with no other constraints. So, for the Friedkin-Johnsen model $\mathcal{Z}$ is the set of all $N \times N$ row-stochastic matrices:

$$\mathcal{Z} = \left\{ N \in [0, 1]^{100 \times 100} \colon \sum_{j=1}^{100} N_{ij} = 1 \text{ for } i = 1,\ 2,\ \ldots,\ 100 \right\}. \tag{7}$$

Additional (e.g., topological) constraints can be embedded within the set $\mathcal{Z}$, for instance by requiring that all nodes have a self-loop, or that the digraph is strongly connected, or has certain average path length, clustering coefficient, or diameter. For the QOL analysis simulations, we require that the digraphs are strongly connected and have a Small-World network topology. Hence, the set of considered underlying digraphs (represented by adjacency matrices) $\mathcal{N} \subset \mathcal{Z}$ is composed of 1000 different strongly connected Small-World network digraphs. Plots showing metrics for these digraphs are presented in Figure S4. The different digraphs were generated by varying the parameters used in the creation of directed Small-World networks, such as the number of edges from the starting Ring digraph, the rewiring probability, and the probability of having bidirectional edges: these variations yield digraphs with the same type of topology, but significantly different metrics, as shown in Figure S4.

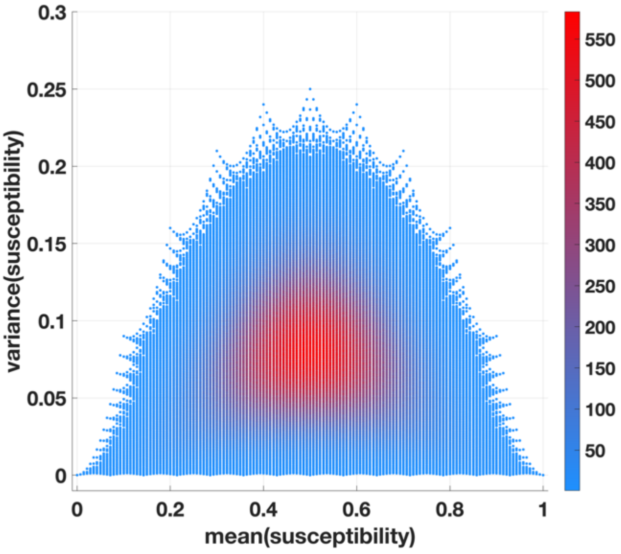

(a) Parameter Plot of the average and the variance of every element in $\mathcal{Y}$. The colour of each dot represents the number of elements in $\mathcal{Y}$ that have the same average and variance.

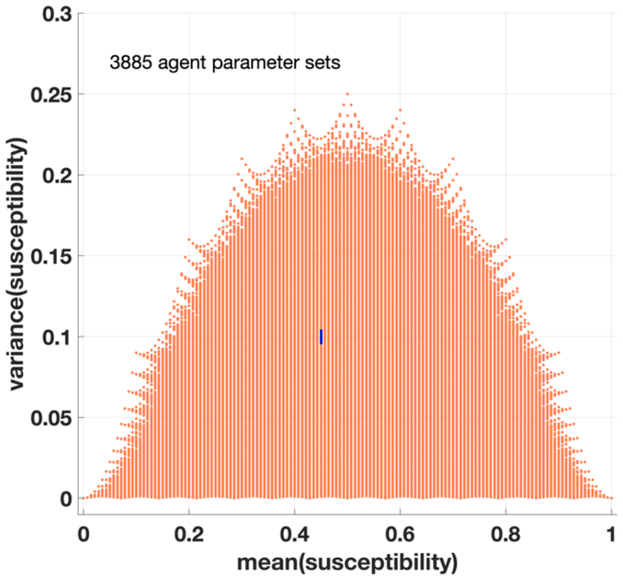

(b) The points highlighted in blue correspond to the set $\mathcal{P}(0.45, 0.1, 0.005)$ according to Equation (6).

Figure S3: Parameter Plot showing average and variance of the susceptibility (agent parameter) sets in the collection $\mathcal{Y}$ according to Equation (5).

For all the results presented in the paper, the set $\mathcal{N}$ contains 1000 digraphs. For the various models, the digraphs are slightly different, because some models use weighted, or signed digraphs. However, for all the considered models, the interconnection topology of the digraphs (namely, the way the vertices are connected by edges, which corresponds to the unsigned and unweighted version of the digraph) remains the same. Precisely, if $\mathcal{N}_{\rm BC}$, $\mathcal{N}_{\rm BEBA}$, $\mathcal{N}_{\rm FJ}$, and $\mathcal{N}_{\rm CB}$ are the sets of digraphs used in the QOL analysis for the Bounded Confidence, Backfire Effect and Biased Assimilation, Friedkin-Johnsen, and Classification-based models, then $|\mathcal{N}_{\rm BC}| = |\mathcal{N}_{\rm BEBA}| = |\mathcal{N}_{\rm FJ}| = |\mathcal{N}_{\rm CB}| = 1000$ and for each digraph in any of these sets, there is one digraph in each of the other sets with the exact same *topology*.

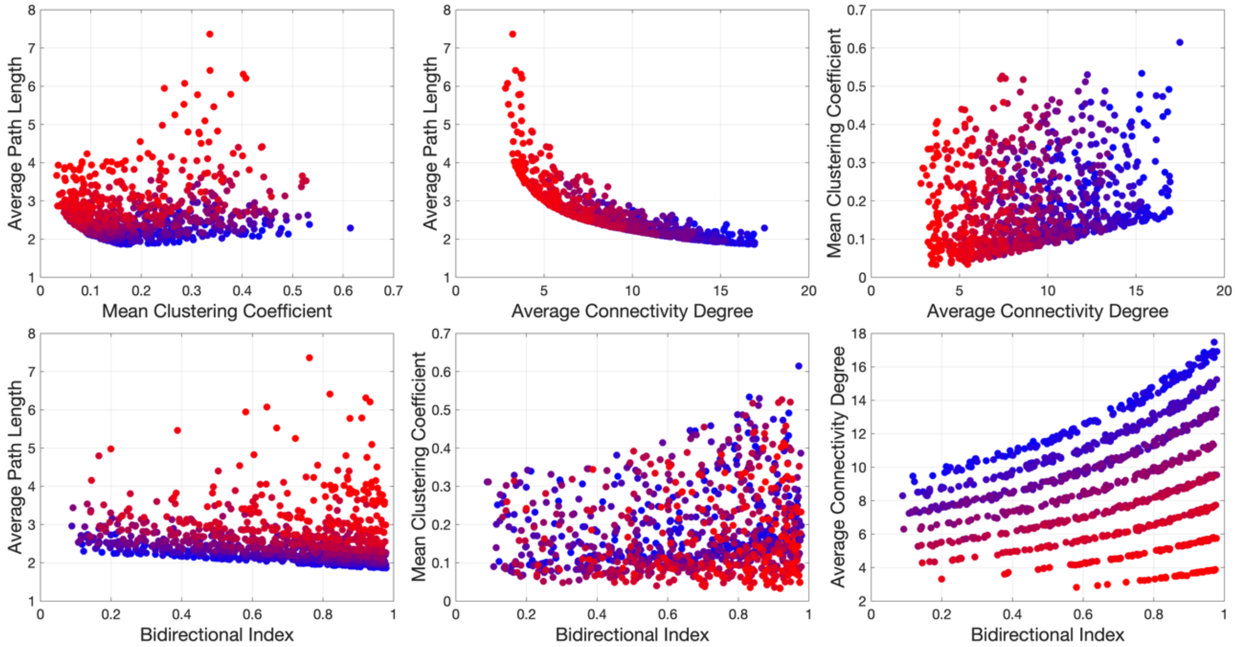

Figure S4: Plots showing the relations between the metrics *average path length*, *clustering coefficient*, *average connectivity degree*, and *bidirectional coefficient*, computed for all the digraphs in the set $\mathcal{N}$. The set $\mathcal{N}$ contains 1000 strongly connected digraphs with Small-World topology, therefore each plot has 1000 points. Points with the same colour correspond to the same digraph. Details on how these topological metrics are computed can be found in Section 5.

The great advantage of approximating the sets $\mathcal{O}$, $\mathcal{P}$, and $\mathcal{N}$ as in Equations (4), (6), and as discussed above is that their cardinality is finite and relatively easy to handle. For instance the collection $\mathcal{O}(0.2, 0.6, 0.05)$ has 52676 elements, as shown in blue in Figure S2b; the collection $\mathcal{P}(0.45, 0.1, 0.005)$ has 3885 elements, as shown in blue in Figure S3b; and the set $\mathcal{N}$ has 1000 elements, with metrics shown in Figure S4. Therefore, choosing one element of these sets with uniform probability can be done simply by ordering the elements and uniformly choosing a random integer in the appropriate interval.

5 Network Metrics

A signed digraph is represented by its weight matrix $W \in \{-1, 0, 1\}^{N\times N}$, where w_{ij} is associated with the edge going from vertex j to vertex i. We consider the following network metrics: average path length (APL), clustering coefficient (CC), average connectivity degree ($\bar{\delta}$), and bidirectional coefficient (BC). This section explains how these metrics are computed.

A directed path is a K-tuple of vertices $(p_1, p_2, \ldots, p_i, p_{i+1}, \ldots, p_K)$ such that there is an edge from vertex p_i to vertex p_{i+1} for $i = 1, \ldots, K-1$. The length $|p|$ of a directed path p is the number of edges that it crosses. Let $P(i, j)$ be the set of all directed paths from vertex i to vertex j (if there are none, then $P(i, j) = \emptyset$). Denote by $d(i, j)$ the length of the shortest directed path from i to j, i.e., $d(i, j) = \min_{p\in P(i,j)} |p|$. Let $C(W)$ be the set of vertex pairs (i, j) such that there exists a direct path from i to j and $i \neq j$, i.e. $C(W) = \{(i, j)\colon P(i, j) \neq \emptyset \text{ and } i \neq j\}$. Then the average path length of the digraph W is:

$$APL = \frac{1}{|C(W)|} \sum_{(i,j)\in C(W)} d(i, j) \tag{8}$$

Note that, because all the networks are strongly connected, $|C(W)| = N(N-1)$.

To compute the average clustering and its variance, consider agent i, with k_i in-neighbours excluding itself: $k_i = |\tilde{\mathcal{N}_i}|$, where $\tilde{\mathcal{N}_i} = \{j \in \mathcal{V}\colon w_{ij} \neq 0,\, i \neq j\}$. Then there are at most $k_i(k_i - 1)$ directed edges between these neighbours. The fraction c_i of these edges that is actually present is the clustering coefficient of agent i. If agent i has only one in-neighbour, then its clustering coefficient is 1, and if it has no in-neighbour but itself c_i is not defined:

$$c_i = \begin{cases} \frac{|\{(j,k)\colon j\neq k \text{ and } i,k\in\tilde{\mathcal{N}_i}\}|}{k_i(k_i-1)} & \text{if } k_i > 1 \\ 1 & \text{if } k_i = 1 \\ \text{undefined} & \text{if } k_i = 0 \end{cases} \tag{9}$$

The average clustering and the clustering variance of the network are thus the average and the variance of the clustering coefficients of all agents with at least one in-neighbour excluding themselves. The average clustering is also sometimes called the clustering coefficient and simply denoted CC (defined by extending to digraphs the definition for undirected graphs by [3]).

For the connectivity degree measures, consider a vertex i. The in-degree (respectively, out-degree) of vertex i is denoted δ^{in} (resp. δ^{out}) and is the number of edges that enter (resp. exit) vertex i. Since each vertex has an individual in- and out-degree it is possible to compute the average and variance of this collection of numbers.

The bidirectional coefficient is computed as the ratio between edges for which an edge connecting the same vertices exists in the opposite direction, and the total number of edges. Mathematically it can be computed by:

$$BC = \frac{\sum_{i\neq j\in\mathcal{V}} |w_{ij}w_{ji}|}{\sum_{i\neq j\in\mathcal{V}} |w_{ij}|} \tag{10}$$

The numerator of Equation (10) counts the number of edges for which there is an edge connecting the same vertices in the opposite direction, and the denominator counts the total number of edges, excluding self-loops.

6 Code usage

The `integerparttable.m` [4] and `intpartgen.m` [5] Matlab scripts by David Holdaway were used as part of the code to create the simulations presented in the paper, to create the sets $\mathcal{O}$ and $\mathcal{P}$.

6.1 Steps to reproduce the results

All simulations were performed using MATLAB. All the code is executed by running the script
`GlobalScript_ProbabilisticAnalysis.m`
There are two ways to do this:

1. Create the figures using the available data. Then, much of the code will not be executed, as the program will simply recreate the figures from existing data. This is of course much faster. To do this, move the `.mat` files contained in the directory `./Resulting_Data` to the main (`PAcode`) directory and run the script
   `GlobalScript_ProbabilisticAnalysis.m`.
2. Create the figures with new data. This will be slower as more computations are needed, in this case, there is no need to modify the contents of the main directory, simply execute the script
   `GlobalScript_ProbabilisticAnalysis.m`
   (this will require 30 cores for parallel computation).

6.2 Datasets

The `GlobalScript_ProbabilisticAnalysis.m` script uses several files that, if not present, are then created.
These are the required files:

- `AllHistograms_10_agents_15_bins.mat`: File that stores information about the sets $\mathcal{X}$ and $\mathcal{Y}$.
- `BC_InitialNetworks_100_agents_1000_networks.mat`: Set $\mathcal{N}$ for the BC model.
- `BCCollection_10_agents_15_bins.mat`: Parameters for the BC model.
- `BEBA_InitialNetworks_100_agents_1000_networks.mat`: Set $\mathcal{N}$ for the BEBA model.
- `BEBACollection_10_agents_15_bins.mat`: Parameters for the BEBA model.
- `CB_InitialNetworks_100_agents_1000_networks.mat`: Set $\mathcal{N}$ for the Classification-based model.
- `CBCollection_10_agents_15_bins.mat`: Parameters for the Classification-based model.
- `FJ_InitialNetworks_100_agents_1000_networks.mat`: Set $\mathcal{N}$ for the Friedkin-Johnsen model.
- `FJCollection_10_agents_15_bins.mat`: Parameters for the Friedkin-Johnsen model.
- `OpinionsCollection_10_agents_15_bins.mat`: Initial opinions

After executing the `GlobalScript_ProbabilisticAnalysis.m` script, these are the resulting datasets that are needed for the `Complete_analysis_new.m` script to create the figures presented in the paper.

- `Results_BC.mat`: Results for the BC model.
- `Results_BEBA.mat`: Results for the BEBA model.
- `Results_CB.mat`: Results for the CB model.
- `Results_FJ.mat`: Results for the FJ model.

References

[1] Devia, C. A. & Giordano, G. A framework to analyze opinion formation models. *Scientific Reports* 1, 13441 (2022).

[2] Wilson, E. Probable inference, the law of succession, and statistical inference. *Journal of the American Statistical Association* 22, 209–212 (1927).

[3] Watts, D. J. & Strogatz, S. H. Collective dynamics of 'small-world' networks. *Nature* 393, 440–442 (1998).

[4] Holdaway, D. integerparttable. Available at https://www.mathworks.com/matlabcentral/fileexchange/36437-integer-partition-generator (2012).

[5] Holdaway, D. intpartgen. Available at https://www.mathworks.com/matlabcentral/fileexchange/36437-integer-partition-generator (2012).

A High Resolution Figures

We provide here larger, high-resolution versions of the figure panels displayed in the main paper.

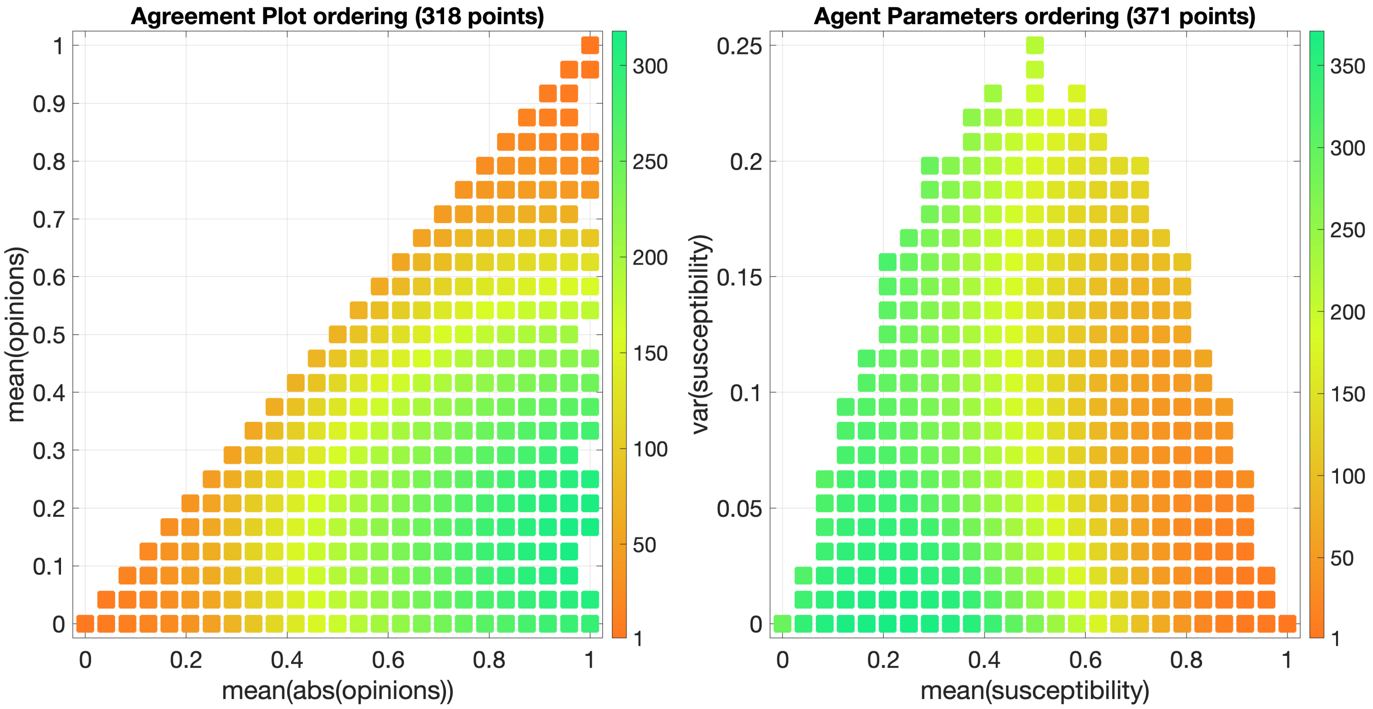

Figure S5: FJ model, with agent parameter sets λ characterised by average $\overline{\lambda}$ and variance $\sigma(\lambda)$.

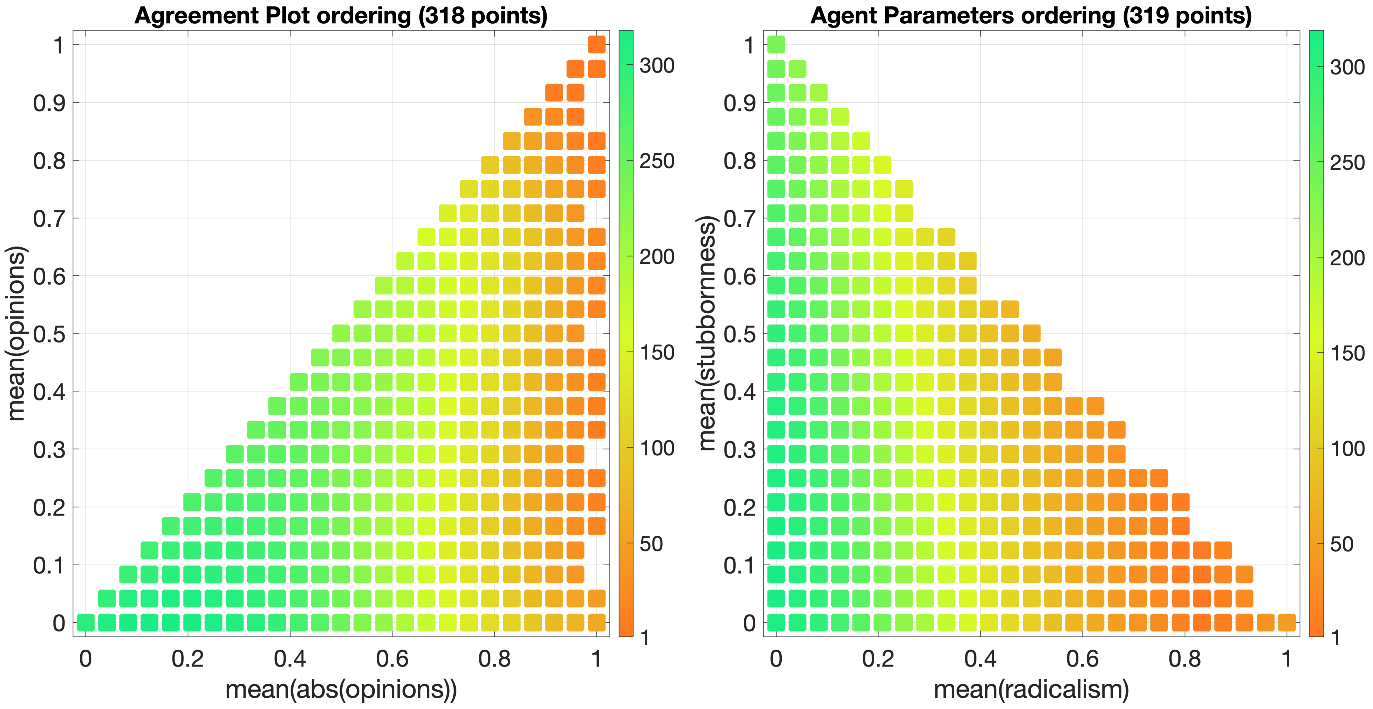

Figure S6: CB model, with agent parameter sets ψ characterised by average radicalism $\overline{\beta}$ and average stubbornness $\overline{\gamma}$.

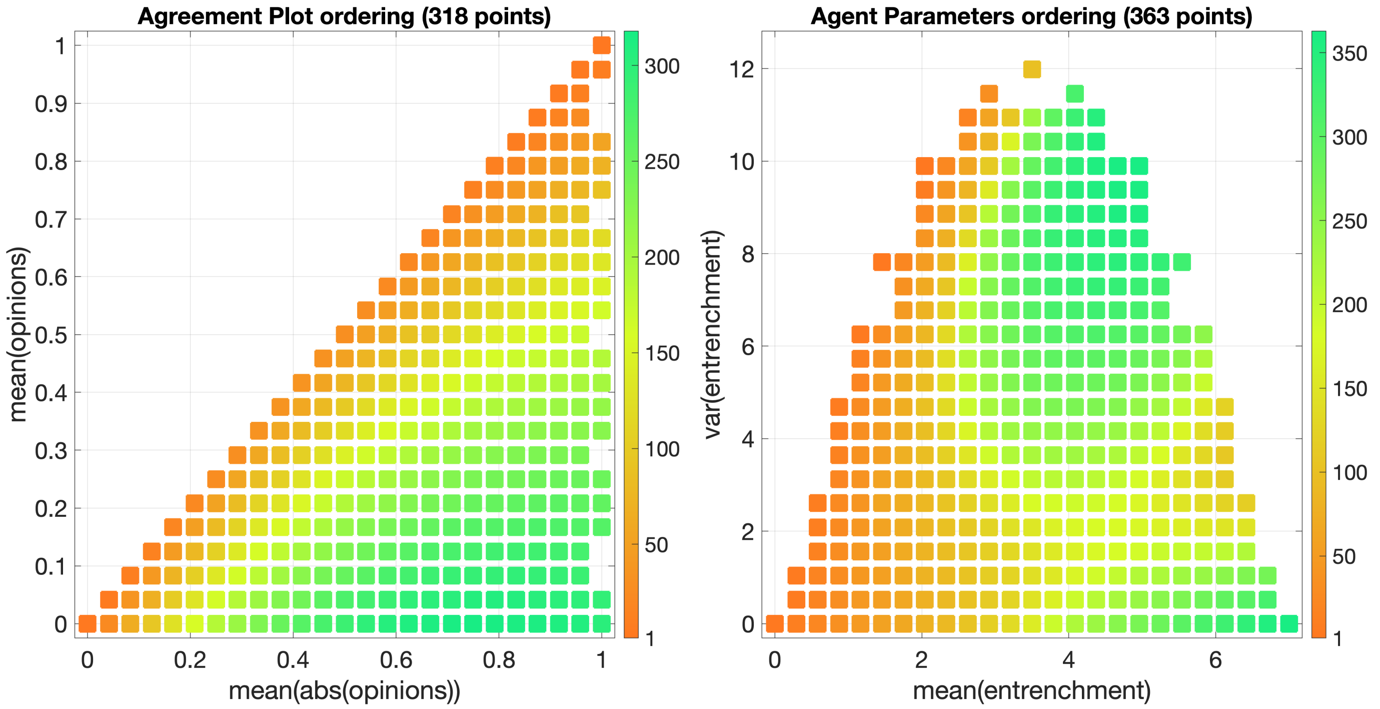

Figure S7: BEBA model, with agent parameter sets ρ characterised by average $\overline{\rho}$ and variance $\sigma(\rho)$.

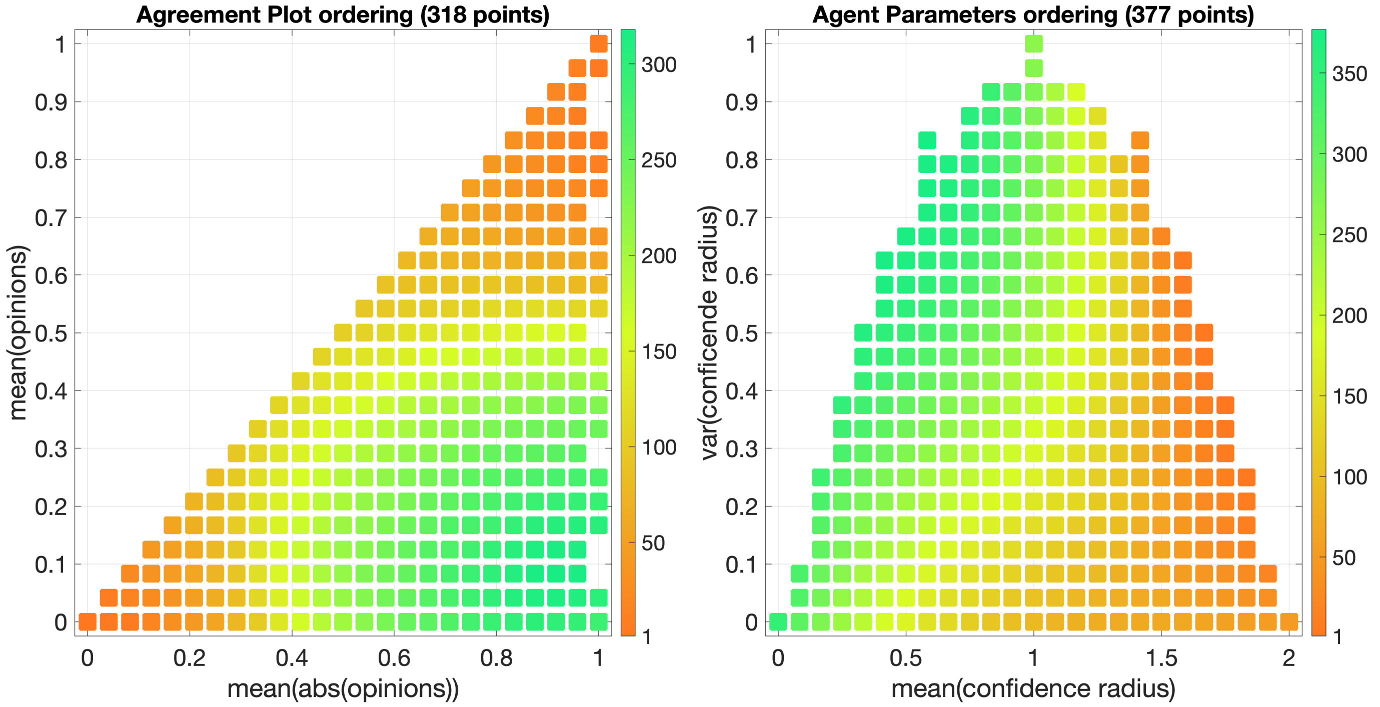

Figure S8: BC model, with agent parameter sets r (confidence radius) characterised by average $\overline{r}$ and variance $\sigma(r)$.

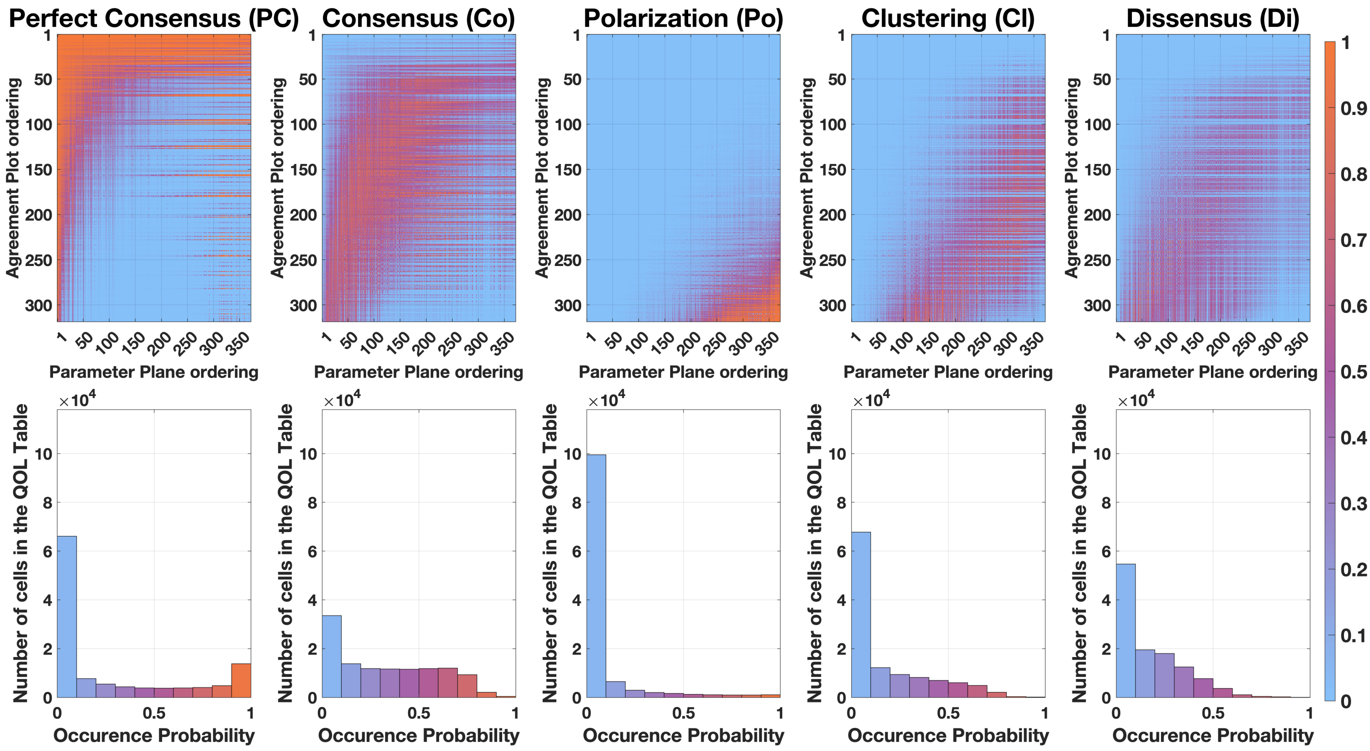

Figure S9: Qualitative Outcome Likelihood Tables FJ model.

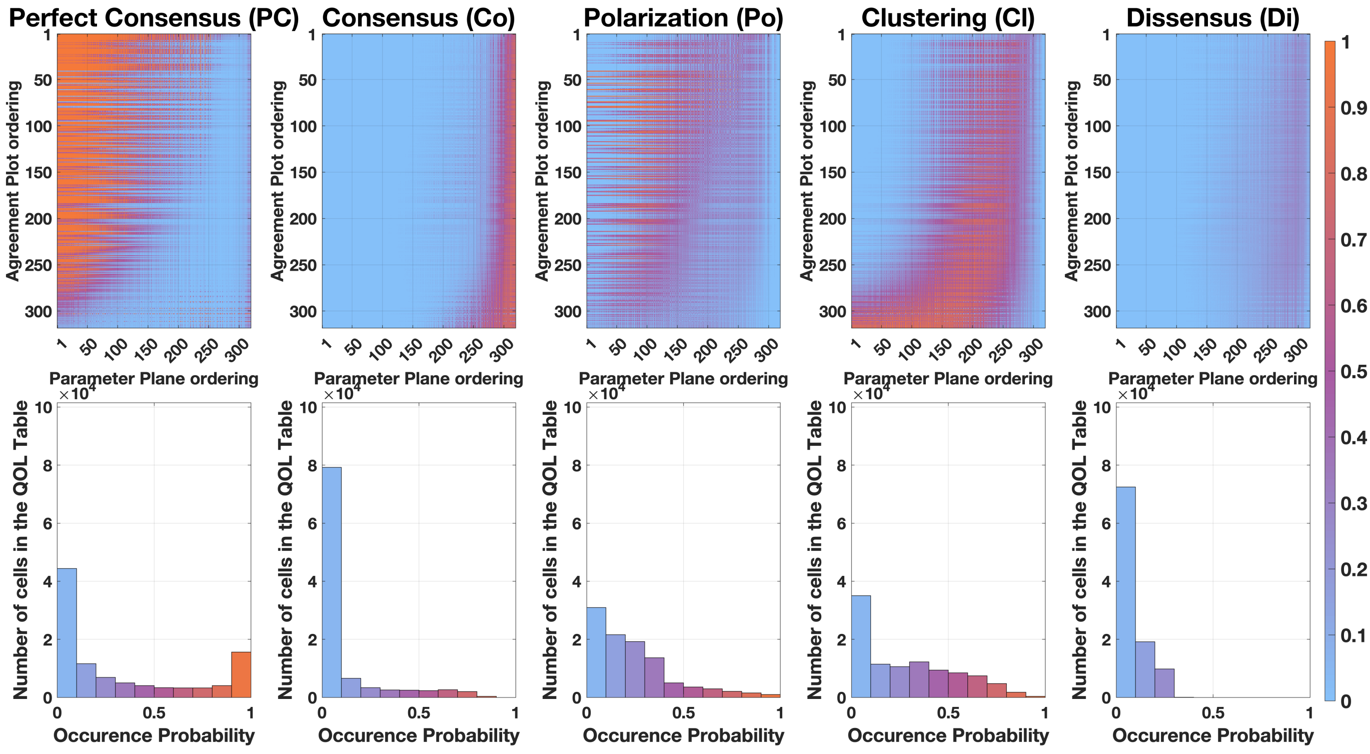

Figure S10: Qualitative Outcome Likelihood Tables CB model.

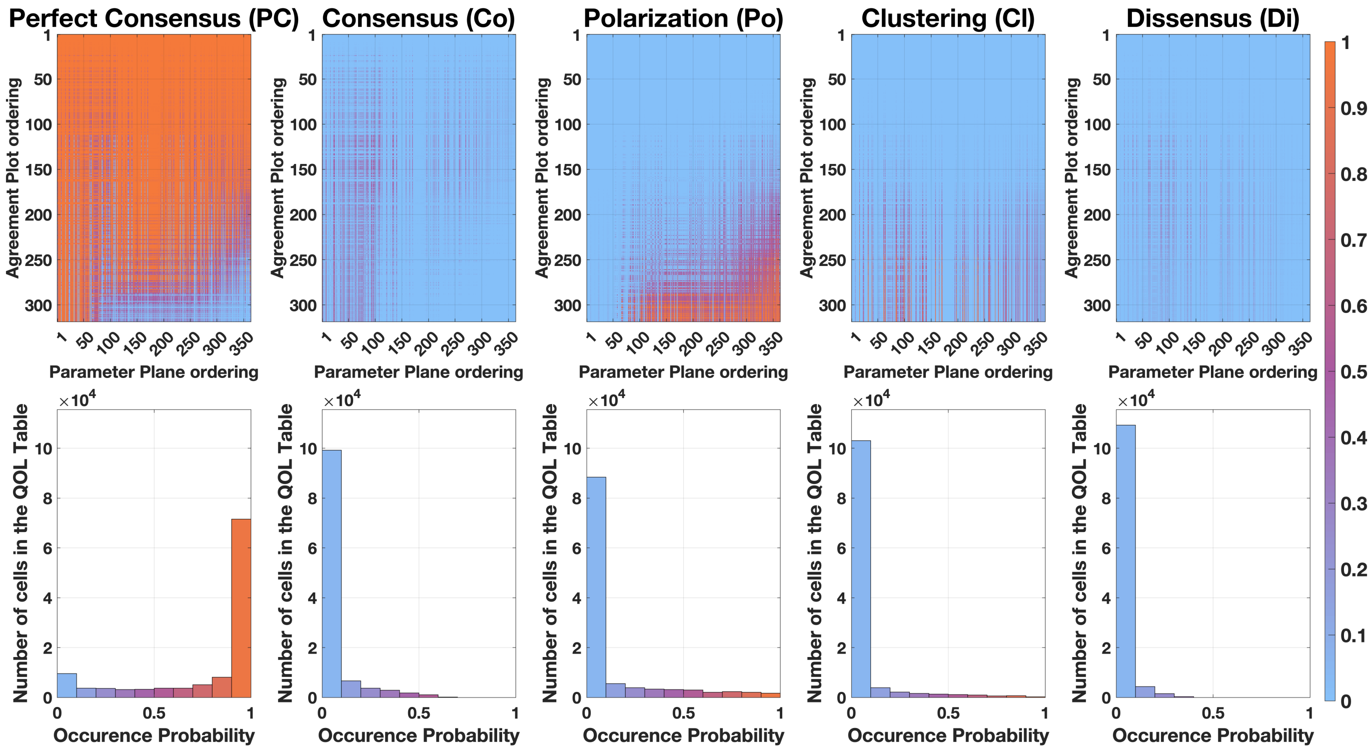

Figure S11: Qualitative Outcome Likelihood Tables BEBA model.

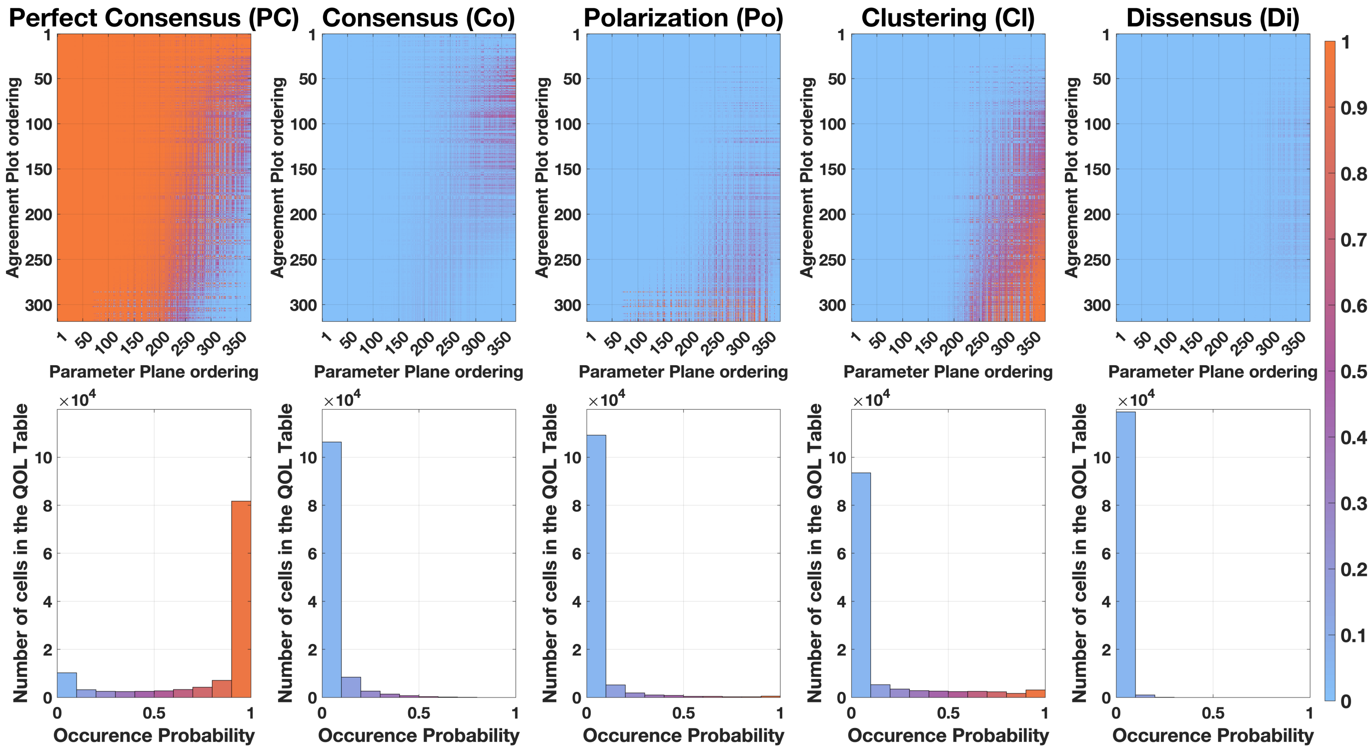

Figure S12: Qualitative Outcome Likelihood Tables BC model.

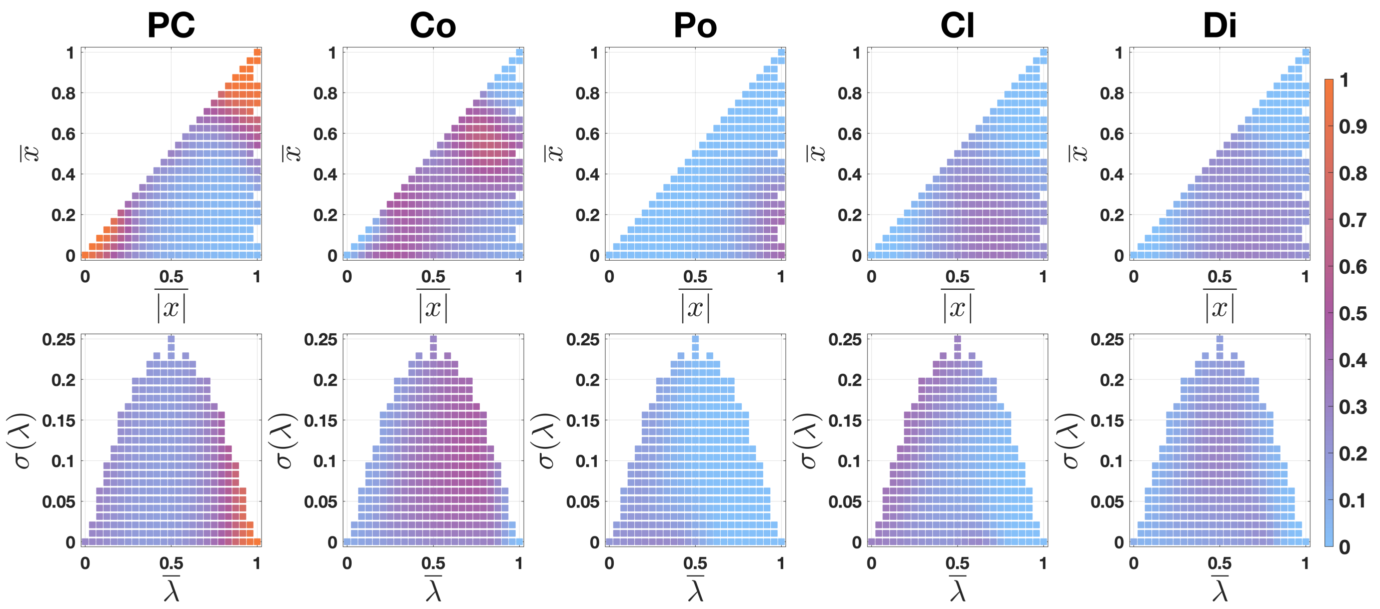

Figure S13: Qualitative Outcome Likelihood Figures FJ model.

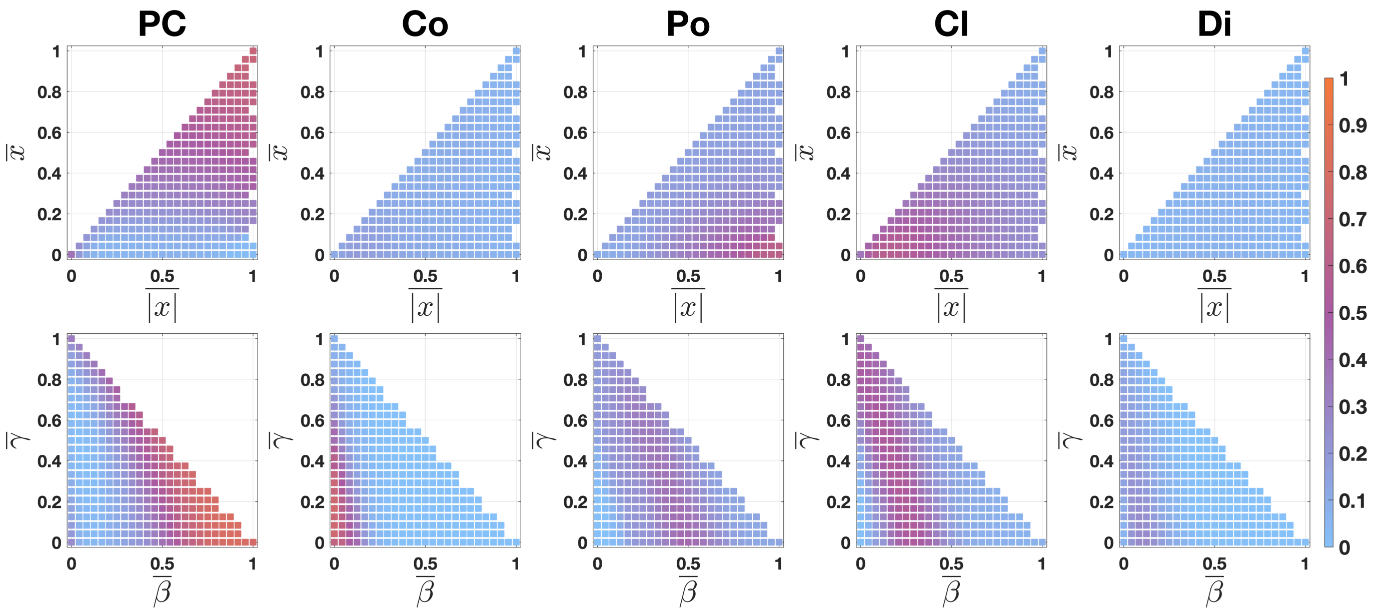

Figure S14: Qualitative Outcome Likelihood Figures CB model.

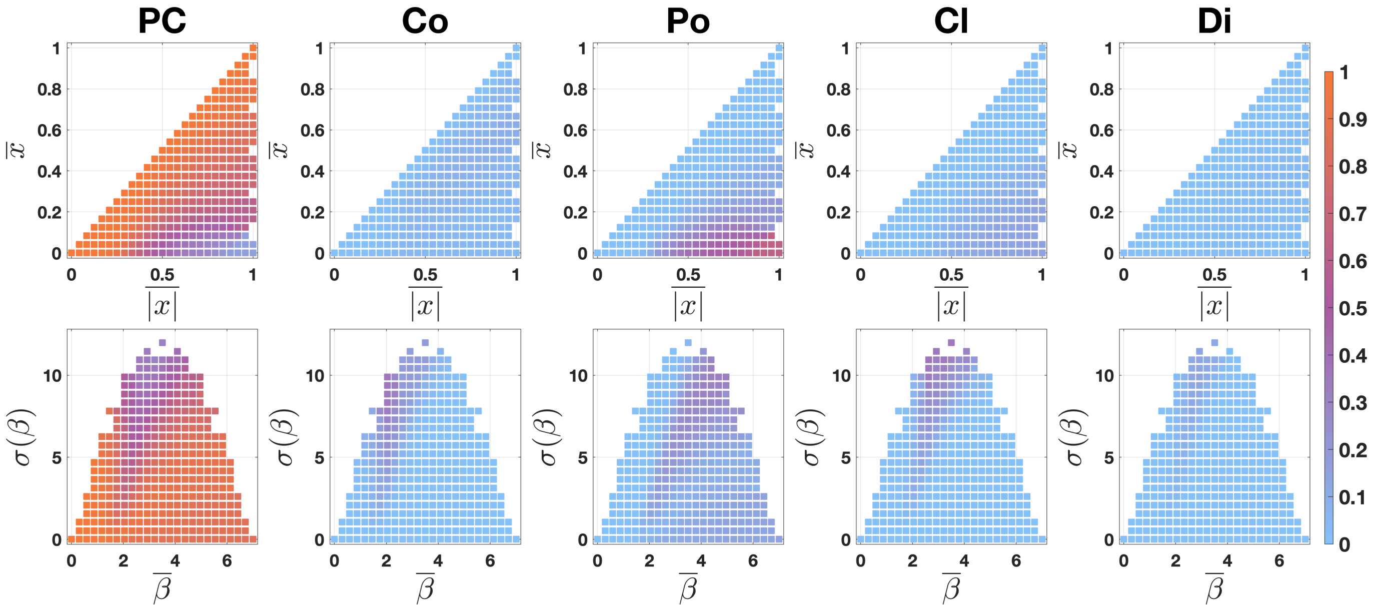

Figure S15: Qualitative Outcome Likelihood Figures BEBA model.

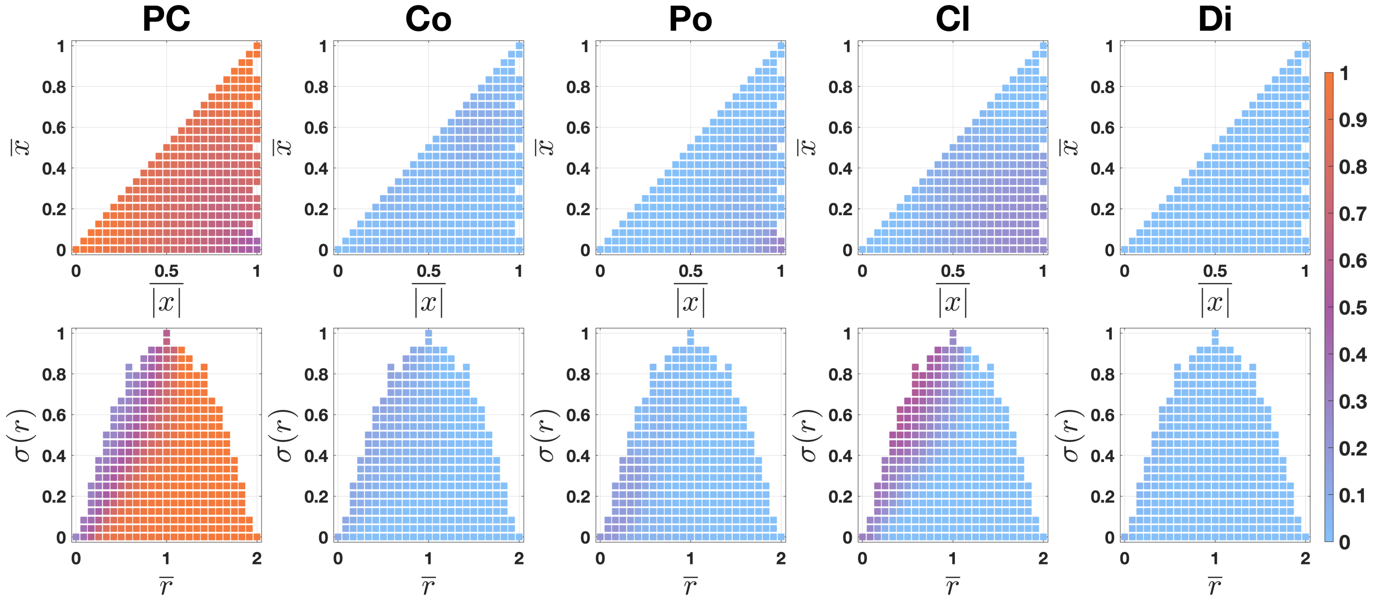

Figure S16: Qualitative Outcome Likelihood Figures BC model.
